# Reciprocal regulation between lunapark and atlastin facilitates ER three-way junction formation

**DOI:** 10.1007/s13238-018-0595-7

**Published:** 2018-11-29

**Authors:** Xin Zhou, Yu He, Xiaofang Huang, Yuting Guo, Dong Li, Junjie Hu

**Affiliations:** 1 Department of Genetics and Cell Biology, College of Life Sciences, Nankai University and Tianjin Key Laboratory of Protein Sciences, Tianjin, 300071 China; 2 National Laboratory of Biomacromolecules, CAS Center for Excellence in Biomacromolecules, Institute of Biophysics, Chinese Academy of Science, Beijing, 100101 China; 3 University of Chinese Academy of Sciences, Beijing, 100101 China

**Keywords:** endoplasmic reticulum, three-way junction, membrane fusion, lunapark, atlastin, amphipathic helix, myristoylation

## Abstract

Three-way junctions are characteristic structures of the tubular endoplasmic reticulum (ER) network. Junctions are formed through atlastin (ATL)-mediated membrane fusion and stabilized by lunapark (Lnp). However, how Lnp is preferentially enriched at three-way junctions remains elusive. Here, we showed that Lnp loses its junction localization when ATLs are deleted. Reintroduction of ATL1 R77A and ATL3, which have been shown to cluster at the junctions, but not wild-type ATL1, relocates Lnp to the junctions. Mutations in the *N*-myristoylation site or hydrophobic residues in the coiled coil (CC1) of Lnp N-terminus (NT) cause mis-targeting of Lnp. Conversely, deletion of the lunapark motif in the C-terminal zinc finger domain, which affects the homo-oligomerization of Lnp, does not alter its localization. Purified Lnp-NT attaches to the membrane in a myristoylation-dependent manner. The mutation of hydrophobic residues in CC1 does not affect membrane association, but compromises ATL interactions. In addition, Lnp-NT inhibits ATL-mediated vesicle fusion *in vitro*. These results suggest that CC1 in Lnp-NT contacts junction-enriched ATLs for proper localization; subsequently, further ATL activity is limited by Lnp after the junction is formed. The proposed mechanism ensures coordinated actions of ATL and Lnp in generating and maintaining three-way junctions.

**Electronic supplementary material:**

The online version of this article (10.1007/s13238-018-0595-7) contains supplementary material, which is available to authorized users.

## Introduction

The ER is composed of tubules and sheets in a continuous membrane system (Chen et al., 2013; Zhang and Hu, 2016). In the cell periphery, the ER exists mostly as a tubular network, with sporadic sheets. Recent super-resolution imaging revealed that some peripheral sheets are fenestrated, and likely still formed by tubule clusters (Nixon-Abell et al., 2016). The cylindrical ER has great physiological significance (Shibata et al., 2006; Bennett, 2012), even though its specific morphological benefits are not entirely clear. The yeast tubular ER proteome suggests that ER tubules mainly play roles in membrane trafficking, lipid synthesis, organellar contacts, and stress signaling (Wang et al., 2017).

ER tubules are constantly pulled out, usually in a microtubule-dependent manner (Du et al., 2004; Grigoriev et al., 2008; Wozniak et al., 2009; Friedman et al., 2010), and subsequently stabilized by integral membrane proteins, namely reticulons and REEPs/Yop1p (Voeltz et al., 2006; Hu et al., 2008). Deletion of these tubule-forming proteins causes a loss of ER tubules and corresponding sheet expansion (Voeltz et al., 2006). Purified tubule-forming proteins, when reconstituted into proteoliposomes, deform the membrane into tubules (Hu et al., 2008; Sun et al., 2016). High curvature seen in the tubule cross-section is generated by wedge insertion and oligomerization-based scaffolding of the proteins (Shibata et al., 2008; Shibata et al., 2009).

A characteristic feature of the peripheral ER is that it contains many three-way junctions as a result of homotypic fusion between tubular membranes. Such fusion is mediated by a class of dynamin-like GTPases called atlastin (ATL) in higher eukaryotes, Sey1p in yeast, and RHD3 in plants (Hu et al., 2009; Orso et al., 2009; Anwar et al., 2012; Zhang et al., 2013). Deletion of ATL results in long unbranched ER tubules indicative of a lack of fusion (Hu et al., 2009). Purified and reconstituted *Drosophila* ATL or Sey1p/RHD3 is capable of merging vesicles *in vitro* (Bian et al., 2011; Anwar et al., 2012; Zhang et al., 2013). These ER fusogens consist of an N-terminal GTPase domain followed by a helix-based stalk region, a transmembrane hairpin, and a C-terminal tail, with both termini facing the cytosol. Structural and biochemical analyses have demonstrated that fusion requires nucleotide-dependent dimerization of the GTPase domain, swing of the stalk triggered by the nucleotide cycle, and continuous GTP hydrolysis (Bian et al., 2011; Yan et al., 2015). An amphipathic helix in the tail of these proteins promotes fusion by destabilizing the lipid bilayer (Liu et al., 2012; Faust et al., 2015).

The shape of a reticular network can be reconstituted *in vitro* using just a tubule-forming protein and a membrane-fusing GTPase (Powers et al., 2017). However, precise regulation of the ER morphology is needed to ensure proper functioning of the ER. A protein family named lunapark (Lnp), which have a conserved “LNPARK” motif, was identified as a regulator of ER shape. Lnp localizes at the three-way junctions. Deletion of Lnp1p in yeast causes unresolved ring closure products, i.e., clusters of junctions (Chen et al., 2012). In mammalian cells, loss of Lnp results in sheet expansion (Shemesh et al., 2014; Chen et al., 2015; Wang et al., 2016) and Lnp-enriched junctions are more stable (Chen et al., 2015; Wang et al., 2016). In plant cells, two homologues of Lnp were identified, which are involved in determining the ER network morphology by regulating the formation of ER cisternae (Kriechbaumer et al., 2018). Lnp possesses two closely spaced transmembrane (TM) segments, forming a TM hairpin (TMH) structure that likely prefers curved membranes, such as ER tubules. The TMH is flanked by an N-terminal cytosolic region (NT) and a C-terminal cytosolic region (CT) that includes a zinc-finger (ZnF) domain. Mammalian Lnp undergoes N-terminal myristoylation (Moriya et al., 2013). The ZnF-containing region forms homotypic associations (Casey et al., 2015; Wang et al., 2016), which may contribute to the negative-edge line curvature seen at the junctions (Shemesh et al., 2014). Phosphorylation in the C-terminus of Lnp, which affects self-association, occurs during mitosis and triggers the expansion of junctional sheets (Wang et al., 2013; Wang et al., 2016). Full length lnp induces the formation of stacks of bicelles *in vitro* through homotypic *in trans* association of C-terminus (Wang et al., 2018). The physiological function of Lnp is largely unclear. In addition to regulating ER morphogenesis, Lnp1p has been proposed to play a role in maintaining nuclear pore complex integrity (Casey et al., 2015) and ER-phagy process (Chen et al., 2018).

Here, we elucidate the mechanism of junctional targeting of Lnp. We show that the NT of Lnp plays a key role, particularly when pairing with junctional enriched ATLs. The proposed mechanism involves recruitment of Lnp by ATL and inhibition of ATL by Lnp.

## Results

### Lnp is recruited to three-way junctions by a subset of ATLs

**Figure 1:**
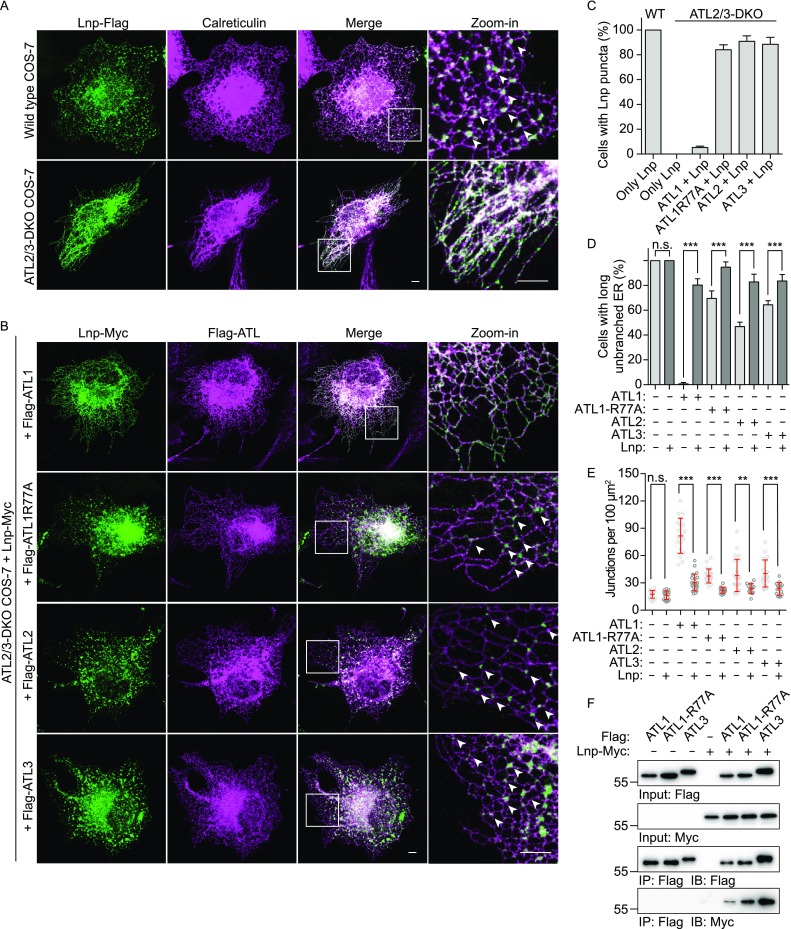
**Localization of Lnp depends on ATL**. (A) Flag-tagged Lnp was expressed in wild type (WT) and ATL-deleted (DKO) COS-7 cells. Localization of Lnp was investigated using anti-Flag antibodies (green). The ER was visualized by indirect immunostaining of endogenous ER luminal marker calreticulin (magenta). (B) Myc-tagged Lnp was co-expressed with Flag-tagged ATLs in ATL-DKO COS-7 cells. Their localizations were determined by anti-Myc (green) and anti-Flag antibodies (magenta). Scale bars = 5 μm. Lnp puncta in (A) and (B) are marked by arrowheads. (C) Percentage of cells with Lnp-enriched puncta in (A) and (B). Columns are shown in mean ± SD. A total of 50 to 60 cells were categorized for each sample. Cells used for counting were pooled from three independent experiments. (D) Percentage of cells with long unbranched ER tubules in (A), (B) and Fig. S1C. Columns are shown in mean ± SD. A total of 50 to 60 cells were categorized for each sample. Cells used for counting were pooled from three independent experiments. NS, no significance; ***P < 0.001 by Fisher’s exact test. (E) Numbers of three-way junctions in tubular ER network per 100 μm^2^ in (A), (B) and Fig. S1C. Data are shown in scatter dot plot with mean ± SD. Numbers of three-way junctions were counted in a total of 15 to 20 random areas from 15 cells in each sample. NS, no significance; **P < 0.01; ***P < 0.001 by Mann-Whitney test. (F) Heterotypic interaction tests between Myc-tagged Lnp and various Flag-tagged ATLs by co-immunoprecipitation (IP). The indicated Flag-ATLs and Lnp-Myc were transfected individually or together into COS-7 cells. Flag-ATLs were precipitated by anti-Flag antibody-conjugated agarose from the cell lysates containing 1% Triton X-100. Samples were immunoblotted (IB) using anti-Myc and anti-Flag antibodies

Previous results suggest that the localization of yeast Lnp1p is regulated by Sey1p (Chen et al., 2012). Therefore, we tested whether mammalian Lnp relies on ATL for proper positioning. As expected, full-length, C-terminal, Flag-tagged human Lnp was enriched at three-way junctions in wild-type COS-7 cells (Fig. 1A). The mammalian ATL family contains three members: ATL1, ATL2 and ATL3 (Zhu et al., 2003). ATL1 is predominantly expressed in the central nervous system, whereas ATL2 and ATL3 are expressed in peripheral tissues (Zhu et al., 2003; Rismanchi et al., 2008). When ATL2 and ATL3, the only ATLs expressed in COS-7 cells, were deleted using the CRISPR/Cas9 system (Fig. S1A) (Sun et al., 2016), Lnp lost its targeting to junctions and became evenly distributed in the tubular ER network (Fig. 1A and 1C). These results suggest that, similar to the yeast counterpart, junction localization of mammalian Lnp depends on ATLs.

Deletion of ATL causes long unbranched ER tubules with fewer three-way junctions (Fig. S1C), suggesting that mis-targeting of Lnp in ATL-deleted cells may be attributed to a loss of junctions. Alternatively, most ATL family members exhibit junction enrichment (Yan et al., 2015), which could physically recruit Lnp. To test whether Lnp localization is directly determined by the presence of ATL or indirectly affected by ER morphology, we expressed different forms of ATL in ATL2/3 double-knock-out (DKO) COS-7 cells and monitored the distribution of Lnp (Fig. 1B, see Fig. S1B for expression levels). The three ATLs have high levels of sequence homology (Zhu et al., 2003). ATL3 is a relatively weaker fusogen and tends to be clustered at the junctions; ATL2 is a stronger fusogen than ATL3 but also enriched at the junctions; ATL1 is the strongest fusogen in the family and usually evenly distributed along ER tubules (Hu et al., 2015; Yan et al., 2015). When ATL1 was expressed in DKO cells, co-expressed Lnp appeared evenly along the ER tubules (Fig. 1B and 1C), even though the morphology of the tubular ER network was mostly normal (Figs. 1D and S1C). In contrast, when ATL2 or ATL3 was expressed, the ER network defects were less well rescued (Figs. 1D and S1C), but when Lnp was co-transfected, its targeting to junctions was mostly restored (Fig. 1B and 1C). Consistently, ATL1 R77A, which possesses reduced GTPase activity and a puncta pattern in the ER, only partially repaired ER morphology (Figs. 1D and S1C) but managed to bring co-expressed Lnp back to junctions (Fig. 1B and 1C). Collectively, these results indicate that the puncta pattern of Lnp is due to the presence of junction-enriched ATLs and less likely by ATL-mediated ER fusion.

Notably, the regulatory role of Lnp in ER morphogenesis was supported by the expression of Lnp not being able to fix ER defects in DKO cells (Fig. 1A and 1D). Similarly, Lnp expression in DKO cells failed to restore amounts of junctions in a given area (Fig. 1E). In contrast, reintroduction of ATLs into DKO cells substantially increased junction counts in cell periphery (Fig. 1E). However, when Lnp was co-transfected, junction number was once again dramatically reduced (Fig. 1E). These results confirm that Lnp is not capable of generating junctions, even though it is known to stabilize junctions (Chen et al., 2015; Wang et al., 2016). In cases where Lnp becomes excessive, it might even prevent junction formation.

To test whether Lnp interacts with ATLs, we performed co-immunoprecipitation experiments. Flag-ATL and Lnp-Myc were co-transfected into COS-7 cells. When Flag-ATL1 R77A or Flag-ATL3 was precipitated by anti-Flag agarose, Lnp-Myc co-precipitated (Fig. 1F). Little but detectable amount of Lnp-Myc was found in precipitates against Flag-ATL1 (Fig. 1F). Reciprocal precipitation was performed with cells transfected only with Lnp-Flag. As expected, endogenous ATL3 co-precipitated with Lnp-Flag (Fig. S2A). These interactions were more prominent when cross-linker DSP was added (Fig. S2B). These results confirm that Lnp interacts with ATLs.

### Lnp-NT is critical for junction localization

**Figure 2:**
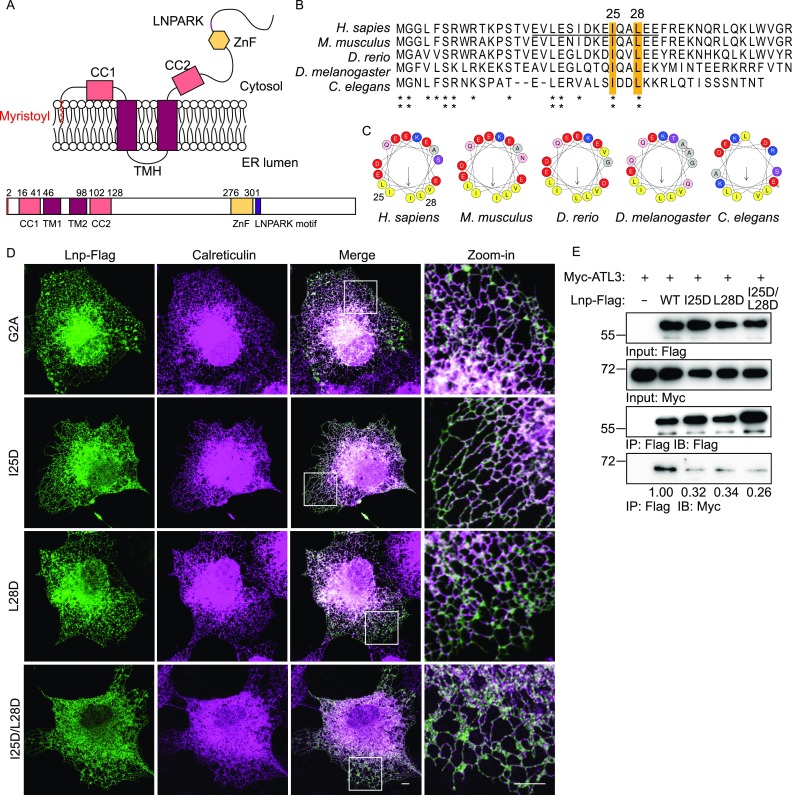
**Lnp-NT regulates the localization of Lnp**. (A) Topology and schematic of the secondary structure of Lnp. CC1, coiled coil 1; TMH, transmembrane hairpin; CC2, coiled coil 2; ZnF, zinc finger. (B) Sequence alignment of Lnp-NT from the indicated species. The amphipathic helix is underlined. Conserved hydrophobic residues I25 and L28 (order number in human Lnp) are highlighted in yellow. *, similar residues; **, identical residues. (C) Helical wheel representation of the underlined sequence in (B) generated using HeliQuest. (D) The indicated mutants of Lnp were expressed in COS-7 cells. Their localization was determined by anti-Flag antibodies (green). The ER was visualized by antibody against ER luminal marker calreticulin (magenta). Scale bars = 5 μm. (E) Interaction tests between Myc-tagged ATL3 and various Flag-tagged LNP mutants by co-immunoprecipitation. Myc-ATL3 was expressed individually (lane 1) or together with Flag-tagged wild-type LNP (lane 2) or the indicated mutants (lanes 3 to 5). Various Lnp-Flag proteins were precipitated from the cell lysates containing 1% Triton X-100 by anti-Flag antibody-conjugated agarose and immunoblotted with anti-Flag and anti-Myc antibodies. Quantification of Co-IPed Myc-ATL3 was performed by Image J

Next, we probed localization determinants in human Lnp. Lnp can be divided into the NT (residues 1–45), TMH (residues 45–96) and CT (residues 97–428) (Fig. 2A). Lnp-NT, which contains an *N*-myristoylation and the first coiled coil (CC1) of Lnp, is mostly conserved among species (Fig. 2B). The first eight residues of Lnp serves as a recognition site for *N*-myristoyltransferase (NMT), which adds a myristoyl group onto G2 after removal of M1. The CC1 is predicted to be an amphipathic helix (Fig. 2C).

When G2 was mutated to alanine, abolishing *N*-myristoylation, Lnp no longer clustered at junctions (Fig. 2D). Similarly, when conserved hydrophobic residues, particularly I25 and L28, were replaced by the charged residue aspartic acid, the mutant proteins became evenly distributed in the ER network (Fig. 2D). In addition, the expression of double mutant I25D/L28D caused drastic defects in ER morphology, including increased Lnp-positive patches in the cell periphery (Figs. 2D and S3A). These results suggest that elements in Lnp-NT play a role in Lnp targeting.

We also tested whether mis-targeting of Lnp-NT mutants is caused by disrupted interactions with ATL. Flag-tagged Lnp and Myc-tagged ATL3 were co-transfected into COS-7 cells. Wild-type or mutant Lnp was precipitated by anti-Flag agarose, and Myc-ATL3 was measured in the precipitates. Mutations at the hydrophobic face of CC1 compromised interactions with ATL3 (Fig. 2E), which could explain their abnormal localization. Interestingly, Lnp G2A increased binding to ATL1 and ATL2 and maintained binding to ATL3 (Fig. S3B). These results suggest that *N*-myristoylation is not essential for ATL association and Lnp can engage different ATLs in different ways.

Proper localization of Lnp may be critical for its regulation of ER morphology. Previous conventional confocal imaging showed that deletion of Lnp in mammalian cells induces sheet expansion (Wang et al., 2016), whereas deletion of Lnp1p in yeast causes the accumulation of junctions (Chen et al., 2012). In light of recent observations made by super resolution microscopy, which suggest that some ER sheets are actually tubular junction clusters (Nixon-Abell et al., 2016), we suspected that the peripheral sheets observed in mammalian Lnp deletion may be clusters of junctions. To this end, we generated Lnp-deleted COS-7 cells using the CRISPR/Cas9 system (Fig. S4A) and performed gazing incidence structural illumination microscopy (GI-SIM). Loss of Lnp led to sheets with uniform signals (Fig. S4B), confirming that Lnp deletion increases peripheral sheets.

**Figure 3:**
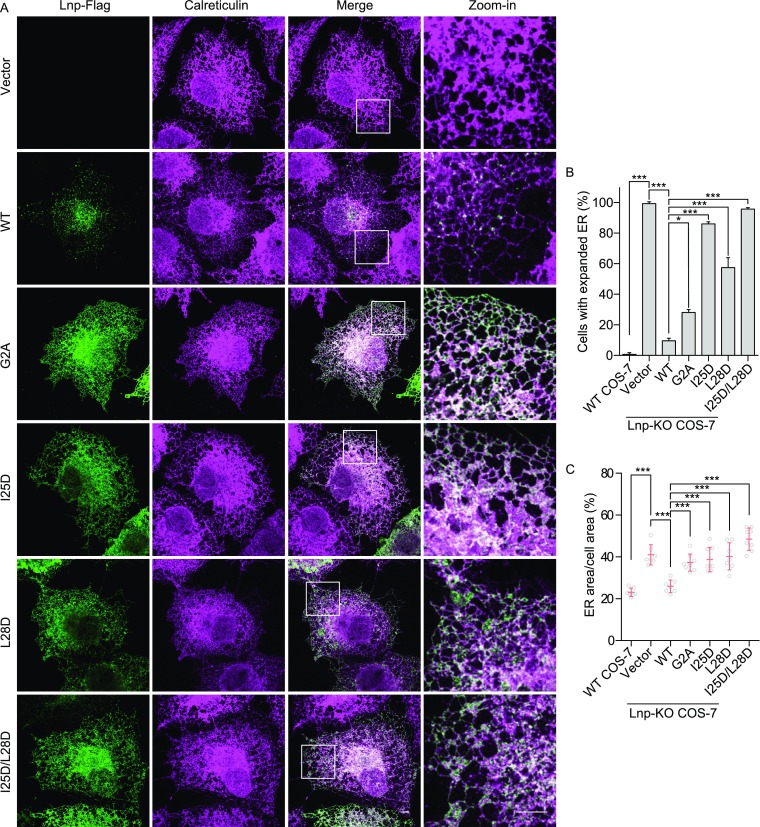
**ER morphology rescue assays in Lnp-deleted COS-7 cells**. (A) Flag-tagged wild-type Lnp and the indicated mutants were expressed in Lnp-deleted (Lnp-KO) COS-7 cells. Their localization was determined by anti-Flag antibodies (green). The ER morphology was visualized by indirect immunostaining of endogenous ER luminal marker calreticulin (magenta). Scale bars = 5 μm. (B) Percentage of cells with expanded ER in (A). Columns are shown in mean ± SD. A total of 50 to 60 cells were categorized for each sample. Cell numbers were counted in three independent experiments. *P < 0.05; ***P < 0.001 by Fisher’s exact test. (C) The ratio of the ER area to the cell area in (A). Data are shown in scatter dot plot with mean ± SD. The cell area and corresponding ER area were measured and analyzed by Image J. 8 to 10 cells in each sample were analyzed. ***P < 0.001 by Mann-Whitney test

We then tested the role of Lnp-NT in regulating the shape of the tubular ER network. As expected, expression of wild-type Lnp in Lnp-deleted COS-7 cells efficiently restored the patterns of the tubular ER network (Fig. 3A and 3B; see Fig. S4C for expression levels). G2A lost its junction localization and its ability to rescue ER morphology was reduced (Fig. 3A and 3B). I25D and L28D were much less active in generating a proper tubular ER network, and the double mutant I25D/L28D nearly failed to do so (Fig. 3A and 3B). We also quantified ER areas in these rescue experiments (Fig. S4D), and found that increases in ER areas correlated with loss of Lnp activity (Fig. 3C). These results suggest that *N*-myristoylation and hydrophobic residues in CC1 are functionally important.

### Lnp-CT is dispensable for junction localization

**Figure 4:**
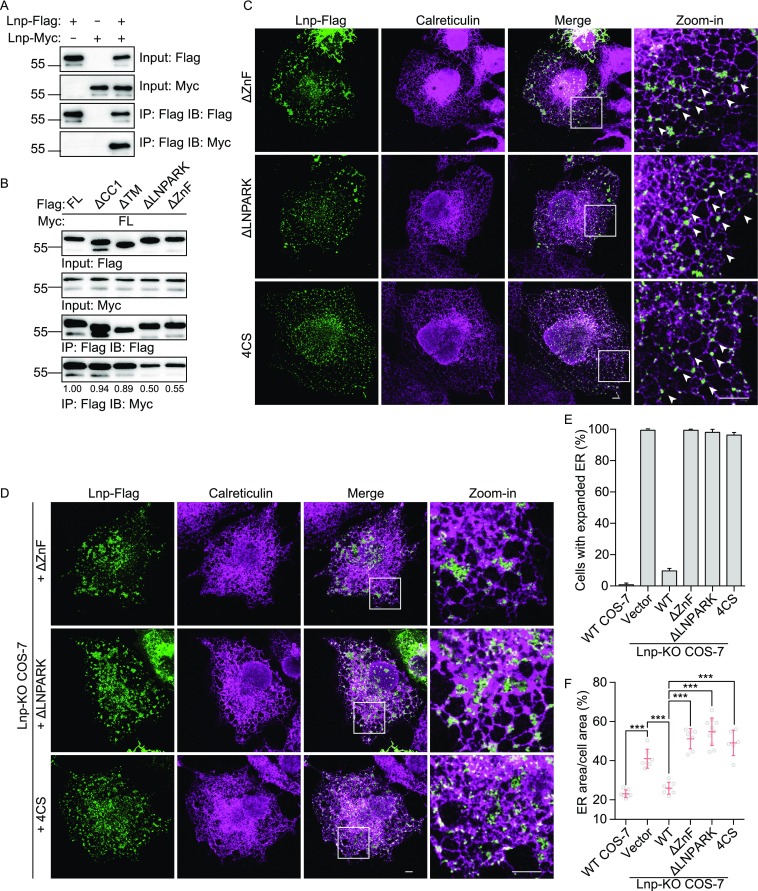
**Impact of Lnp-CT on its localization**. (A) Homotypic interaction of Lnp by co-immunoprecipitation. Flag- and Myc-tagged wild-type Lnp were expressed individually or together in COS-7 cells. Lnp-Flag was precipitated by anti-Flag antibody-conjugated agarose from cell lysates containing 1% Triton X-100. Samples were immunoblotted with anti-Flag and anti-Myc antibodies. (B) As in (A), but with wild-type Myc-tagged Lnp co-expressed with various truncations in COS-7 cells. Quantification of co-IPed Myc-Lnp was performed by Image J. (C) The indicated Flag-tagged constructs with CT mutant were expressed in wild-type COS-7 cells. Their localization was investigated using anti-Flag antibodies (green). ER morphology was visualized by indirect immunostaining of endogenous ER luminal marker calreticulin (magenta) Lnp puncta are marked by arrowheads. (D) As in (C), but with the indicated constructs expressed in Lnp-KO COS-7 cells. Scale bars = 5 μm. (E) Percentage of cells with expanded ER in (D). Columns are shown in mean ± SD. A total of 50 to 60 cells were categorized for each sample. Cell numbers were counted in three independent experiments. (F) The ratio of the ER area to the cell area in (D). Data are shown in scatter dot plot with mean ± SD. The cell area and corresponding ER area were measured and analyzed by Image J. 8 to 10 cells in each sample were analyzed. ***P < 0.001 by Mann-Whitney test

After analyzing the role of Lnp-NT in localization, we moved onto Lnp-CT. The CT of Lnp possesses CC2 (residues 102–128) and a ZnF domain (residues 276–301) (Fig. 2A). CC2 has been speculated to complement CC1, and the ZnF domain has been shown to mediate homotypic oligomerization (Casey et al., 2015; Wang et al., 2016). To identify key features of Lnp-CT, we performed co-immunoprecipitation. Consistently, full-length Lnp self-associated, as judged by co-precipitation of Lnp-Flag and Lnp-Myc (Fig. 4A). Lnp-TM is not necessary because full-length Lnp also precipitated LnpΔTM, and Lnp-NT is less important because full-length Lnp still efficiently interacted with LnpΔCC1 (Fig. 4B). Conversely, deletion of ZnF in the CT reduced homotypic interactions (Fig. 4B). These results confirm that the CT, particularly the ZnF domain, plays a dominant role in mediating the homotypic interactions of Lnp.

The “LNPARK” motif, which gives rise to the protein’s name, immediately follows the ZnF domain (Fig. S5A). Structural modeling of the ZnF region revealed that the LNPARK motif folds as a β-strand and forms anti-parallel interactions with another β-strand within the ZnF (Fig. S5B). When the LNPARK motif was deleted in full-length Lnp, the loss of self-association was equivalent to ZnF deletion (Fig. 4B). These data indicate that LNPARK likely participates in the proper folding of the ZnF and indirectly regulates the oligomerization of Lnp.

To assess the involvement of the CT in Lnp localization, we transfected various CT mutants into COS-7 cells and monitored their localization. Deletion of the entire ZnF or just the LNPARK motif did not change the junction enrichment of Lnp (Fig. 4C), suggesting that self-oligomerization is dispensable for Lnp localization. Consistent with these findings, substitution of the four conserved cysteines in the ZnF domain with serines had little impact on Lnp localization (Fig. 4C). We also tested whether CT-mediated oligomerization is important for ER morphogenesis. When three CT mutants were transfected individually into Lnp-deleted cells, none of them were able to generate the proper shape of the tubular ER network (Fig. 4D and 4E; see Fig. S5C for expression levels). Consistently, these Lnp mutants failed to reduce ER expansion caused by Lnp deletion (Fig. 4F).

The ZnF self-association suggests that a soluble CT fragment may act in a dominant negative manner. When the CT was expressed in wild-type COS-7 cells, it generally appeared as a diffuse pattern in the cytosol. However, the ER underwent sheet expansion as in Lnp deletion (Fig. S5D and S5E). The dominant negative defects were diminished when the ZnF was removed from the CT (Fig. S5D and S5E). Furthermore, CC2, the other structural element in the CT, is not needed for Lnp oligomerization, as CTΔCC2 caused changes to the ER morphology in COS-7 cells (Fig. S5D and S5E). Consistently, double deletion of CC2 and the ZnF in the CT resulted in the same behavior as CTΔZnF (Fig. S5D and S5E). Taken together, the results indicate that the ZnF in the CT is needed for the maintenance of proper ER morphology but not for the localization of Lnp.

### Lnp-NT attaches to membranes

**Figure 5:**
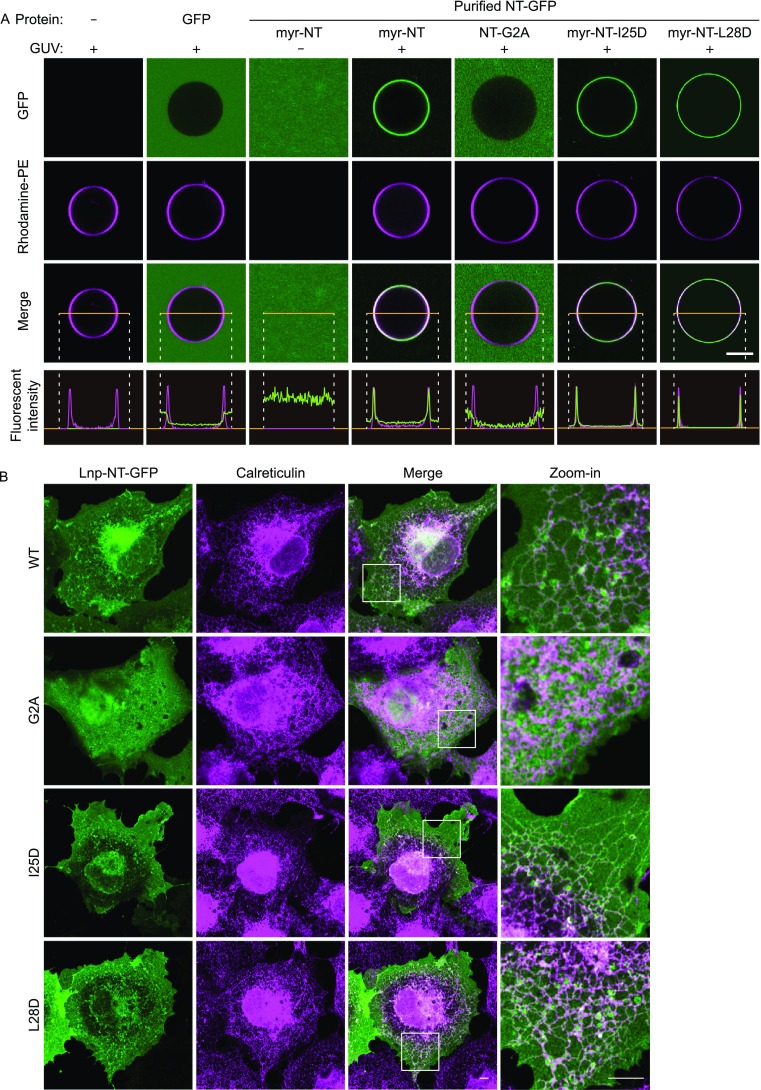
***N*-myristoylation anchors Lnp-NT to membranes**. (A) *In vitro* GUV binding assays using the indicated purified C-terminal GFP tagged Lnp-NT constructs. In the line-scanning analysis along the yellow segments, the fluorescence intensity of various GFP-tagged Lnp-NTs (green) was compared to the corresponding rhodamine-labeled GUVs (magenta). (B) The indicated C-terminal EGFP-tagged Lnp-NT constructs were expressed in COS-7 cells. Localization of Lnp-NT visualized by EGFP signal (green). The ER was visualized by indirect immunostaining of endogenous ER luminal marker calreticulin (magenta). Scale bars = 5 μm

To further analyze the molecular activity of Lnp, we purified Lnp-NT from *E. coli* in the presence of *N*-myristoyltransferase (NMT) and myristic acid. Modification of Lnp-NT was confirmed by mass spectrometry using purified protein (Fig. S6A). The *N*-myristoylation is expected to occur in membranes. In addition, the amphipathic nature of CC1 would suggest that it could be inserted into the lipid bilayer. Therefore, we tested whether the NT associates with membranes. When GFP-tagged Lnp-NT was incubated with rhodamine-labeled giant unilaminar vesicles (GUVs), GFP signals clearly highlighted the surface of the GUV (Fig. 5A). This was not seen with GFP only. The proximity of Lnp-NT and the membrane was confirmed by FRET signals between GFP and rhodamine (Fig. S6B). Substitution of G2 with alanine, which abolishes myristoylation, disrupted the GUV association of Lnp-NT. In contrast, mutations in the hydrophobic face of CC1, including I25D and L28D, did not change the membrane attachment (Fig. 5A). These results suggest that Lnp-NT can directly bind to membranes via *N*-myristoylation, but not CC1.

Liposome flotation experiments provided further evidence of an interaction of Lnp-NT with lipids. Proteins and liposomes were mixed thoroughly and placed in the bottom of a sucrose gradient. Upon centrifugation, the liposomes float to the top of the gradient and interaction with the liposomes can be judged by co-migration of proteins to the top. As expected, some myristoylated Lnp-NT appeared in the top fractions of liposomes (Fig. S6C). Prevention of *N*-myristoylation in G2A mutant blocked flotation of the protein, but mutation of I25 or L28 did not. Next, we tested the membrane attachment of Lnp-NT in a cellular context. GFP-tagged Lnp-NT was transfected into wild-type COS-7 cells. Most of the green fluorescent signals were diffused in the cytosol, but some of them appeared at ER tubules (Fig. 5B), as indicated by calreticulin, a commonly used ER marker. The weak co-localization was retained with I25D and L28D, but diminished with G2A mutant (Fig. 5B). Collectively, these results confirm that *N*-myristoylation anchored Lnp-NT in ER membranes with little help from CC1.

### Lnp-NT inhibits ATL-mediated fusion

When Lnp and ATLs were co-expressed in ATL-deleted cells, we noticed that the rescue of ER morphological defects by ATL was drastically affected with the co-overexpression of Lnp (Fig. 1D). Thus, we tested whether purified Lnp-NT could inhibit ATL-mediated vesicle fusion *in vitro*. Because only *Drosophila* ATL (dmATL) exhibits prominent fusion activity *in vitro*, we first measured cross-species Lnp-ATL interactions. While dmATL exhibited junctional enrichment as previously reported (Fig. S7A), dmLnp failed to cluster at junctions in wild type COS-7 cells (Fig. S7B and S7D), likely because endogenous monkey ATLs do not efficiently bind to dmLnp. Only when dmATL and dmLnp were co-transfected into COS-7 cells, dmLnp became mostly puncta (Fig. S7C and S7D). Similarly, dmLNP was not able to replaced monkey Lnp in Lnp-deleted COS-7 cells (Fig. S7E). It was reported that expression of ATL could partly compensate the loss of Lnp (Wang et al., 2016). However, dmATL failed to rescue Lnp deletion in COS-7 cells. Restoration of ER morphology was only seen when dmATL and dmLnp were co-expressed (Fig. S7E). These results suggest that dmATL pairs more efficiently with dmLnp than other Lnp.

**Figure 6:**
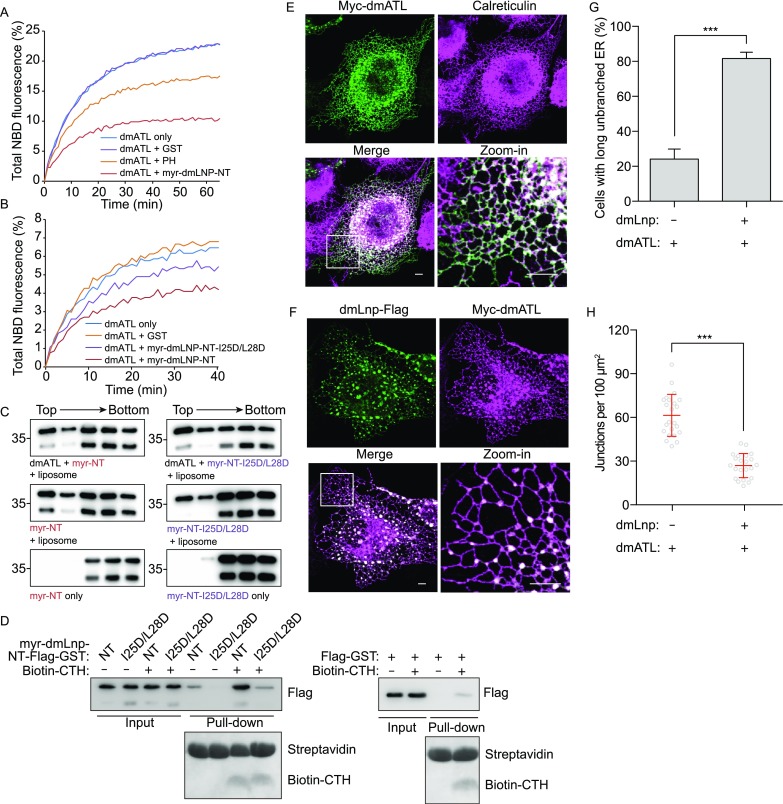
**Lnp-NT inhibits ATL activity**. (A) Full-length Drosophila dmATL was purified and reconstituted into donor and acceptor vesicles at a 1:2,000 protein-to-lipid ratio. The GTP-dependent fusion of donor and acceptor vesicles was monitored by the dequenching of a 7-nitro-2-1,3-benzoxadiazol-4-yl (NBD)-labeled lipid included in the donor vesicles. Purified Drosophila dmLnp-NT and a PH domain from HT-008 were 5-times the concentration of dmATL. (B) As in (A), but with a protein-to-lipid ratio of 1:3,000. The dmLnp-NT with the I25D/L28D double-mutant was compared to wild-type dmLnp-NT. In both (A) and (B), GST was used as a negative control. (C) Proteoliposomes containing the indicated C-terminal tandem GST-Flag-tagged Drosophila wild-type dmLnp-NT and I25D/L28D mutant were floated in a sucrose gradient and the fractions analyzed by immunoblotting using anti-Flag antibody. (D) Biotin-labeled CTH was pulled down by streptavidin-conjugated agarose. Bounded purified Lnp-NT-Flag-GST or I25D/L28D mutant was examined by immunoblotting (IB) with anti-Flag antibodies. Flag-GST was used as negative control. Streptavidin and CTH were used as loading controls. (E) Myc-tagged drosophila dmATL was expressed in ATL-DKO COS-7 cells. Localization of dmATL was determined by anti-Myc antibody (green). The ER was visualized by indirect immunostaining of endogenous ER luminal marker calreticulin (magenta). (F) Flag-tagged dmLnp was co-expressed with Myc-tagged dmATL in ATL-DKO COS-7 cells. Their localization was determined by anti-Flag (green) and anti-Myc antibodies (magenta). Scale bars = 5 μm. (G) Percentage of cells with long unbranched ER tubules in (E) and (F). Columns are shown in mean ± SD. A total of 50 to 60 cells were categorized for each sample. Cell numbers were counted in three independent experiments. ***P < 0.001 by Fisher’s exact test. (H) Numbers of three-way junctions in tubular ER network per 100 μm^2^ in (E) and (F). Data are shown in scatter dot plot with mean ± SD. Numbers of three-way junctions were counted in a total of 23 random areas from 23 cells in each sample. ***P < 0.001 by Mann-Whitney test

We purified dmLnp-NT with a C-terminal GST tag. In a previously established fusion assay, ATL was reconstituted into both donor vesicles containing NBD- and rhodamine-labeled PE at quenching concentrations, and acceptor vesicles, to which no labeled PE was added. The merging of lipids between two types of vesicles can be measured by the dequenching of NBD upon the enlargement of membrane areas. When myristoylated Lnp-NT was included, the fusion activity of ATL was reduced to ~50% of samples without Lnp (Fig. 6A). The inhibition was not seen with GST alone, and to a lesser extent with an irrelevant membrane-interacting PH domain, suggesting that the effect is less likely caused by membrane crowdedness. When I25 and L28 were both mutated, ATL inhibition was drastically reduced (Fig. 6B). As expected, myristoylated dmLnp showed association with both liposome and dmATL-reconstituted proteoliposomes (Fig. 6C). Same results were obtained with I25D/L28D double mutant, even though the membrane-attached mutant likely decreased its ATL interaction (Fig. 6C). ATL uses hydrophobic residues of an amphipathic helix (APH) in its CT to disturb the lipid bilayer and facilitate fusion process (Liu et al., 2012). We speculated that membrane attached Lnp-NT engages the APH in ATL-CT and subsequently inhibits fusion activity of ATL. To test this hypothesis, we performed *in vitro* pull-down experiments using biotin-labeled dmATL CTH peptide and purified Lnp-NT. As predicted, wild type Lnp-NT was successfully co-precipitated with the peptide, but the I25D/L28D mutant was not (Fig. 6D). Collectively, these results indicate that dmLnp-NT is capable of preventing dmATL-mediated fusion; when it is myristoylated, the interaction and subsequent inhibition involves the hydrophobic face of CC1.

To confirm the inhibition of dmLnp on dmATL in cells, we performed ER morphology rescue assay in DKO cells. Similar to human ATLs, dmATL efficiently restored the pattern of the tubular ER network (Fig. 6E and 6G), as descried previously (Wu et al., 2015). When dmLnp was co-expressed with dmATL (Fig. S7F), the ability of dmATL to rescue ER morphology was significantly reduced (Fig. 6F and 6G). In agreement with the morphological changes, the amount of junctions was elevated in the presence of dmATL and dropped to that of untransfected DKO cells after the addition of dmLnp (Fig. 6H). These results indicate that dmLnp inhibits the activity of dmATL in cells.

## Discussion

**Figure 7:**
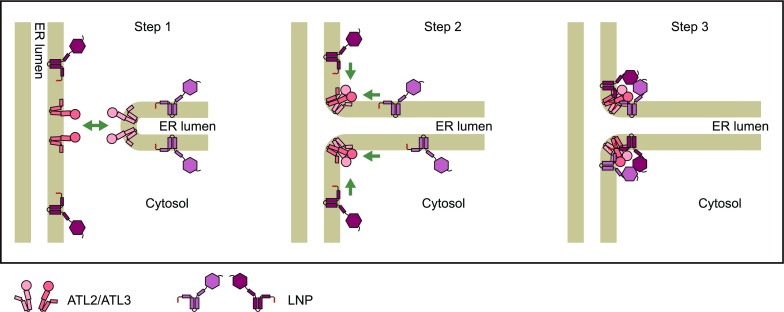
A model for generating three-way junctions. See “DISCUSSION” for details

Our results reveal how precisely Lnp and junctional enriched ATL cooperate to generate three-way junctions (Fig. 7). First, ATL molecules from apposing membrane tubules tether and fuse membranes by GTP hydrolysis. Merged membranes create a newly formed three-way junction. Retained ATLs at the junction then attract Lnp from surrounding tubular membranes. Finally, junction-enriched Lnp reduces the activity of interacting ATLs to prevent further unnecessary action, stabilizing the junction.

We show that the N-terminus of Lnp dips into membranes using *N*-myristoylation. Because CC1 is followed by TMH, it is likely pinned onto the lipid bilayer by flanking membrane anchors. Although expected to partially insert into membranes as an amphipathic helix, CC1 actually uses its hydrophobic face to contact ATL. As a result, ATL-mediated fusion can be reduced. We previously demonstrated that a conserved amphipathic helix exists in many dynamin-like fusogenic GTPases. This helix inserts shallowly into the outer leaflet of the lipid bilayer and prepares membranes for subsequent lipid mixing. When considering potential interacting regions in ATL by Lnp-CC1, it is reasonable to juxtapose CC1 with APH in ATL. If such an interaction reorients the hydrophobic face of ATL-APH, it would explain the inhibitory effect of Lnp on ATL. Given that Lnp-NT lies on lipid bilayer in this mode of ATL engagement, we termed it “NT-down” state of Lnp.

Our results also indicate that some ATL family members can interact with Lnp in yet to be discovered ways. For example, ATL1 and ATL2 bind Lnp much better when Lnp is incapable of *N*-myristoylation, but ATL3 is insensitive to the G2A mutation. However, all these ATLs can be inhibited by Lnp, suggesting that the alternative ATL-Lnp interactions are likely inhibitory for ATL activity. Such interactions may explain why Lnp is still functional in cells that only express ATL1, even though the characteristic puncta pattern of Lnp is lost in these conditions. In addition, the *N*-myristoylation site in Lnp of high eukaryotes is not conserved in yeast Lnp1p, even though the junctional targeting of Lnp1p depends on Sey1p. It is proposed that Lnp1p antagonizes Sey1p activity. If a direct impact does exist, it would occur when the NT of Lnp1p is free from membrane anchoring, thus the “NT-up” state. It is possible that Lnp switches between the up and down conformation; some ATLs may rely on just one mode of Lnp engagement, whereas other ATLs are regulated by Lnp in at least two ways.

Proper localization of Lnp is critical for its role in the maintenance of the tubular ER network. Though, Lnp-CT has little influence on Lnp localization, it is indispensable for ER morphogenesis, likely by mediating oligomerization. Reconstitution of purified full-length *Xenopus* Lnp with phospholipids forms stacked membrane bicelle discs, which is mediated by *in trans* interactions between Lnp-CT sitting in apposing discs. It is found that the unstructured segment with multiple phosphorylated sites, thus called P domain, prior to ZnF plays an important role in stacking (Wang et al., 2018). Our results demonstrate the role of the LNPARK motif as a structural complement of the ZnF and suggest that ZnF also participates in homotypic association. A ZnF is commonly identified in E3 ligase, and the Lnp-NT has been reported to exhibit E3 activity (Zhao et al., 2016). Combining purified Lnp-NT with a few promiscuous E2s, we failed to observe self-ubiquitination. The possibility of ubiquitin-dependent regulation thus requires further investigation. In addition to maintaining the shape of the ER, Lnp has been found to interact with COPII-related components (McGourty et al., 2016) and appear on the surface of lipid droplets (Krahmer et al., 2013), suggesting additional functions to be discovered.

## Materials and methods

### Plasmid construction

#### Mammalian cell expression constructs

For mammalian gene constructs, C-terminal Flag- and Myc-tagged wild-type human Lnp were PCR amplified and inserted into pcDNA4T/O (Invitrogen). The regions coding Lnp-NT (1–45) and CT (99–428) were generated from wild-type Lnp construct. NT was fused to the EGFP tag. Mutants G2A, I25D, L28D, I25D/L28D and 4CS were generated by site-directed mutagenesis from wild-type Lnp construct. Truncations ΔCC1, ΔZnF and ΔLNPARK were generated by overlap-PCR. CTΔZnF, CTΔCC2 and CTΔCC2ΔZnF were generated from the Lnp-CT construct. Myc-ATL1, Flag-ATL1, Myc-ATL2, Flag-ATL2, Myc-ATL3, Flag-ATL3 and Flag-ATL1R77A were described previously (Hu et al., 2009; Yan et al., 2015). Codon-optimized dmATL and dmLnp were amplified and inserted into pcDNA4T/O as described previously (Wu et al., 2015). mEmerald-Calreticulin was a gift from Dong Li’s lab.

#### *E. coli* expression constructs

For protein purification constructs, the C-termini of codon-optimized human Lnp-NT (1–45) and *Drosophila* dmLnp-NT (1–41) were fused with GST/GFP and Flag tags in tandem and inserted into pET28a. Point mutations in human Lnp-NT, including G2A, I25D, L28D and I25D/L28D, were generated by site-directed mutagenesis from wild-type human Lnp-NT. *Drosophila* dmLnp-NT point mutant I25D/L28D was generated by site-directed mutagenesis from wild-type dmLnp-NT.

### Cell culture and transfection

Cells were incubated in DMEM (Gibco) containing 10% fetal bovine serum (Gibco) at 37 °C in 5% CO_2_ and passaged every 2–3 days. Transient transfections were performed using Lipofectamine 3000 (Invitrogen) or X-tremeGENE™ HP DNA Transfection Reagent (Roche) following the manufacturers’ instructions. For immunoprecipitation and Western blot, 70% confluent cells were transfected and harvested 24 h later. For immunofluorescent imaging, 30% confluent cells were transfected and fixed 16–24 h later.

### Gene knock-out using CRISPR/Cas9

Lnp knock-out in COS-7 cells was generated by CRISPR/Cas9-mediated genome editing using pX330 vector (Addgene). The guide RNA (gRNA) used for Lnp knock-out was GTGGATTATTTTCTCGATGG, which was described previously (Wang et al., 2016). Briefly, cells were transfected with pX330 coding the gRNA targeting Lnp and isolated as single cell colonies 24 h later. After several passages, colonies were harvested to verify the Lnp expression level by Western blot. ATL2 and ATL3 double knock-out COS-7 cell stain was described previously (Sun et al., 2016).

### Antibodies

For Western blot, primary antibodies were anti-LNP (rabbit, Sigma), anti-Flag (mouse, Sigma), anti-Myc (rabbit, Sigma) and anti-β-actin (mouse, Sigma). Secondary antibodies were goat anti-rabbit IgG-peroxidase antibody and goat anti-rabbit IgG-peroxidase antibody (Sigma).

For immunofluorescent staining, primary antibodies were anti-Flag (mouse, Sigma), anti-Myc (rabbit, Sigma), anti-Myc (mouse, Santa Cruz), and anti-calreticulin (rabbit, Abcam). Secondary antibodies were goat anti-rabbit IgG Alexa Fluor®555, donkey anti-mouse IgG Alexa Fluor®488, donkey anti-rabbit IgG Alexa Fluor®488 and donkey anti-mouse IgG Alexa Fluor®594 (Invitrogen).

### Immunofluorescent staining and imaging

As described (Zhu et al., 2018), cells cultured on coverslips were washed with PBS twice, fixed with 4% paraformaldehyde (PFA) dissolved in 1× PBS for 25 min, permeabilized with 0.1% Triton X-100 in 1× PBS for 10 min and blocked with 3% bovine serum albumin (BSA) in 1× PBS for 1 h. Samples were incubated with primary antibodies (diluted 1:500) overnight at 4 °C, and then with secondary antibodies (diluted 1:800) for 1 h at room temperature. Finally, the coverslips were sealed with glycerol mounting medium containing anti-fade reagent. Unless otherwise stated, images were captured on a Leica TCS SP5 confocal microscope with a 63×/1.40 N.A.Plan Apochromat oil immersion objective lens using LAS AF (version 1.3.1 build 525) software. High-resolution fluorescent images were captured by GI-SIM.

### Immunoprecipitation

For immunoprecipitation, cell lysates were prepared with 1 mL lysis buffer (50 mmol/L Tris (pH 7.5), 150 mmol/L NaCl, 1% indicated detergents (Triton X-100 or digitonin), and protease inhibitor (Roche)). Flag-tagged proteins were incubated with EZview™ Red ANTI-FLAG® M2 Affinity Gel (Sigma) agarose for 2 h at 4 °C. The agarose was then washed with IP buffer (50 mmol/L Tris (pH 7.5), 150 mmol/L NaCl, 0.1% indicated detergent and protease inhibitor) four times. Precipitated proteins were eluted by 2× SDS-PAGE sample buffer and detected by Western blot.

### Protein expression and purification

All proteins were purified from *E*. *coli* strain Transetta (Transgene). Transformed bacteria were grown in LB medium with 50 μg/mL kanamycin to an OD_600_ of 0.8 at 37 °C, and then induced with 0.3 mmol/L isopropyl-β-_D_-thiogalactoside for 14 h at 28 °C. For proteins needing myristoylation, 5 μmol/L myristic acid was added to the LB medium 10 min before induction. Bacteria were harvested by centrifugation at 4,000 rpm. Each 1 g cell pellet was suspended in 10 mL buffer A (50 mmol/L Tris (pH 8.0), 145 mmol/L KCl, 5 mmol/L NaCl, 2 mmol/L MgCl_2_, 2 mmol/L DTT, 5% glycerol and 1 mmol/L phenylmethanesulfonyl fluoride) and lysed by sonication. The lysates were clarified by centrifugation at 38,900 ×*g* for 1 h. Human recombinant Lnp-NTs were first isolated using an Ni-NTA column (GE Healthcare) in buffer A containing 10 mmol/L imidazole and eluted with buffer A containing 250 mmol/L imidazole. *Drosophila* recombinant dmLnp-NTs were first isolated using a Glutathione Sepharose 4 Fast Flow column (GE Healthcare) in buffer A and eluted by buffer A containing 10 mmol/L reduced glutathione. All proteins were further purified by gel filtration chromatography using Superdex 200 Increase10/300GL (GE Healthcare) in buffer A. Fractions containing the protein of interest were pooled and concentrated. Samples were verified by Coomassie brilliant blue staining.

Full-length *Drosophila* ATL was purified from *E*. *coli* strain BL21 as a GST fusion protein as described previously (Orso et al., 2009). Bacteria were harvested by centrifugation. The cell pellet was washed once with 25 mmol/L Hepes (pH 7.5) and 200 mmol/L KCl, and lysed through a microfluidizer in buffer A200 (25 mmol/L Hepes (pH 7.5), 200 mmol/L KCl, 2 mmol/L EDTA, 2 mmol/L β-mercaptoethanol, 10% glycerol). Membranes were pelleted by centrifugation at 42,000 rpm using the Type 45 Ti rotor (Beckman Coulter). The membrane pellet was solubilized in buffer A200 with 4% Triton X-100 for 20 min at 4 °C. The extract was centrifuged and the protein bound to glutathione Sepharose. After elution, the protein was passed through a PD-10 column (GE Healthcare) to remove the glutathione. The GST tag was cleaved from ATL by thrombin. Samples were verified by Coomassie brilliant blue staining.

### Lipid preparation and GUV binding assays

Giant unilamellar vesicles contain 64% PC, 15% DOPS, 19.5% POPE and 1.5% rhodamine-DPPE (Avanti). GUVs were made by electro-formation. Briefly, a lipid mix in chloroform was deposited on indium-titan oxide glass slides and dried for 30 min in a vacuum to evaporate all solvents. GUVs were electroformed in 2 mL of 400 mmol/L sucrose at 3 V and 10 Hz for 4 h at 55 °C.

Purified GFP, human Lnp-NT-GFP, Lnp-NT-G2A-GFP, Lnp-NT-I25D-GFP and Lnp-NT-L28D-GFP were mixed with GUVs in a protein-to-lipid molar ratio of 1:500. The mixtures were softly mixed at 40 rpm on a horizon rotator at room temperature for 90 min before imaging by confocal microscopy.

### Floatation assays

The mixture of liposome and purified protein (30 μL) was mixed with 100 μL of 1.9 mol/L sucrose overlaid with 100 μL 1.25 mol/L sucrose and 20 μL buffer without sucrose. The samples were centrifuged in a Beckman rotor TLS 55 at 174,000 ×*g* and 4 °C for 90 min. The gradient was fractionated into five 50 μL fractions and verified by Western blot.

### Pull-down assays

Biotin-labeled synthetic peptide corresponding to helix in dmATL C-terminal tail was described previously (Liu et al., 2012). Peptides were bounded to streptavidin-conjugated agarose (Sigma), and then incubated with purified dmLnp-NT-Flag-GST for 40 min at 4 °C. The agarose was then washed with buffer A four times. Precipitated dmLnp-NT were eluted by 2× SDS-PAGE sample buffer and detected by Western blot. Streptavidin and imported peptides were measured by coomassie blue staining.

### Fusion assays

Preformed donor and acceptor liposomes were prepared as described previously (Orso et al., 2009) and the reconstitution and fusion assay performed (Bian et al., 2011). A protein-to-lipid molar ratio of 1:2,000 to 1:3,000 was used in both donor and acceptor liposome vesicles. To assess the inhibition of fusion by dmLnp-NT, purified NT and NT mutants were mixed with dmATL proteoliposomes. The molar ratio of dmLnp-NT to dmATL was 5:1. All fusion reactions were performed in parallel in 50 μL volumes and started by the addition of GTP to a final concentration of 1 mmol/L. All data were calibrated by setting to zero at 0 min and subtracting the baseline values obtained in the absence of GTP. All data were expressed as a percentage of the max fluorescence observed upon adding 10 μL of 2.5% dodecylmaltoside to the reactions after 60 min.

### Image processes and statistics

Image transformation and quantification were conducted by ImageJ version 1.49. Data were subjected to statistical analysis and plotted using Graphpad Prism version 6.0c. Comparisons were performed using the non-parametric Mann-Whitney test or Fisher’s exact test. For all analyses, a *P*-value < 0.05 was considered significant. Brightness and contrast were adjusted across the entire image using Adobe Photoshop CC 2015.

## Electronic supplementary material

Below is the link to the electronic supplementary material.

Supplementary material 1 (PDF 3742 kb)

## References

[CR1] Anwar K, Klemm RW, Condon A, Severin KN, Zhang M, Ghirlando R, Hu J, Rapoport TA, Prinz WA (2012). The dynamin-like GTPase Sey1p mediates homotypic ER fusion in *S. cerevisiae*. J Cell Biol.

[CR2] Bennett PM (2012). From myofibril to membrane; the transitional junction at the intercalated disc. Front Biosci (Landmark Ed).

[CR3] Bian X, Klemm RW, Liu TY, Zhang M, Sun S, Sui X, Liu X, Rapoport TA, Hu J (2011). Structures of the atlastin GTPase provide insight into homotypic fusion of endoplasmic reticulum membranes. Proc Natl Acad Sci USA.

[CR4] Casey AK, Chen S, Novick P, Ferro-Novick S, Wente SR (2015). Nuclear pore complex integrity requires Lnp1, a regulator of cortical endoplasmic reticulum. Mol Biol Cell.

[CR5] Chen S, Cui Y, Parashar S, Novick PJ, Ferro-Novick S (2018). ER-phagy requires Lnp1, a protein that stabilizes rearrangements of the ER network. Proc Natl Acad Sci USA.

[CR6] Chen S, Desai T, McNew JA, Gerard P, Novick PJ, Ferro-Novick S (2015). Lunapark stabilizes nascent three-way junctions in the endoplasmic reticulum. Proc Natl Acad Sci USA.

[CR7] Chen S, Novick P, Ferro-Novick S (2012). ER network formation requires a balance of the dynamin-like GTPase Sey1p and the Lunapark family member Lnp1p. Nat Cell Biol.

[CR8] Chen S, Novick P, Ferro-Novick S (2013). ER structure and function. Curr Opin Cell Biol.

[CR9] Du Y, Ferro-Novick S, Novick P (2004). Dynamics and inheritance of the endoplasmic reticulum. J Cell Sci.

[CR10] Faust JE, Desai T, Verma A, Ulengin I, Sun TL, Moss TJ, Betancourt-Solis MA, Huang HW, Lee T, McNew JA (2015). The Atlastin C-terminal tail is an amphipathic helix that perturbs the bilayer structure during endoplasmic reticulum homotypic fusion. J Biol Chem.

[CR11] Friedman JR, Webster BM, Mastronarde DN, Verhey KJ, Voeltz GK (2010). ER sliding dynamics and ER-mitochondrial contacts occur on acetylated microtubules. J Cell Biol.

[CR12] Grigoriev I, Gouveia SM, van der Vaart B, Demmers J, Smyth JT, Honnappa S, Splinter D, Steinmetz MO, Putney JW, Hoogenraad CC (2008). STIM1 is a MT-plus-end-tracking protein involved in remodeling of the ER. Curr Biol.

[CR13] Hu J, Shibata Y, Voss C, Shemesh T, Li Z, Coughlin M, Kozlov MM, Rapoport TA, Prinz WA (2008). Membrane proteins of the endoplasmic reticulum induce high-curvature tubules. Science.

[CR14] Hu J, Shibata Y, Zhu PP, Voss C, Rismanchi N, Prinz WA, Rapoport TA, Blackstone C (2009). A class of dynamin-like GTPases involved in the generation of the tubular ER network. Cell.

[CR15] Hu X, Wu F, Sun S, Yu W, Hu J (2015). Human atlastin GTPases mediate differentiated fusion of endoplasmic reticulum membranes. Protein Cell.

[CR16] Krahmer N, Hilger M, Kory N, Wilfling F, Stoehr G, Mann M, Farese RV, Walther TC (2013). Protein correlation profiles identify lipid droplet proteins with high confidence. Mol Cell Proteomics.

[CR17] Kriechbaumer V, Breeze E, Pain C, Tolmie F, Frigerio L, Hawes C (2018). Arabidopsis Lunapark proteins are involved in ER cisternae formation. New Phytol.

[CR18] Liu TY, Bian X, Sun S, Hu X, Klemm RW, Prinz WA, Rapoport TA, Hu J (2012). Lipid interaction of the C terminus and association of the transmembrane segments facilitate atlastin-mediated homotypic endoplasmic reticulum fusion. Proc Natl Acad Sci USA.

[CR19] McGourty CA, Akopian D, Walsh C, Gorur A, Werner A, Schekman R, Bautista D, Rape M (2016). Regulation of the CUL3 ubiquitin ligase by a calcium-dependent co-adaptor. Cell.

[CR20] Moriya K, Nagatoshi K, Noriyasu Y, Okamura T, Takamitsu E, Suzuki T, Utsumi T (2013). Protein N-myristoylation plays a critical role in the endoplasmic reticulum morphological change induced by overexpression of protein Lunapark, an integral membrane protein of the endoplasmic reticulum. PLoS ONE.

[CR21] Nixon-Abell J, Obara CJ, Weigel AV, Li D, Legant WR, Xu CS, Pasolli HA, Harvey K, Hess HF, Betzig E (2016). Increased spatiotemporal resolution reveals highly dynamic dense tubular matrices in the peripheral ER. Science.

[CR22] Orso G, Pendin D, Liu S, Tosetto J, Moss TJ, Faust JE, Micaroni M, Egorova A, Martinuzzi A, McNew JA (2009). Homotypic fusion of ER membranes requires the dynamin-like GTPase atlastin. Nature.

[CR23] Powers RE, Wang S, Liu TY, Rapoport TA (2017). Reconstitution of the tubular endoplasmic reticulum network with purified components. Nature.

[CR24] Rismanchi N, Soderblom C, Stadler J, Zhu PP, Blackstone C (2008). Atlastin GTPases are required for Golgi apparatus and ER morphogenesis. Hum Mol Genet.

[CR25] Shemesh T, Klemm RW, Romano FB, Wang S, Vaughan J, Zhuang X, Tukachinsky H, Kozlov MM, Rapoport TA (2014). A model for the generation and interconversion of ER morphologies. Proc Natl Acad Sci USA.

[CR26] Shibata Y, Hu J, Kozlov MM, Rapoport TA (2009). Mechanisms shaping the membranes of cellular organelles. Annu Rev Cell Dev Biol.

[CR27] Shibata Y, Voeltz GK, Rapoport TA (2006). Rough sheets and smooth tubules. Cell.

[CR28] Shibata Y, Voss C, Rist JM, Hu J, Rapoport TA, Prinz WA, Voeltz GK (2008). The reticulon and DP1/Yop1p proteins form immobile oligomers in the tubular endoplasmic reticulum. J Biol Chem.

[CR29] Sun S, Lv L, Yao Z, Bhanot P, Hu J, Wang Q (2016). Identification of endoplasmic reticulum-shaping proteins in Plasmodium parasites. Protein Cell.

[CR30] Voeltz GK, Prinz WA, Shibata Y, Rist JM, Rapoport TA (2006). A class of membrane proteins shaping the tubular endoplasmic reticulum. Cell.

[CR31] Wang S, Powers RE, Gold VA, Rapoport TA (2018). The ER morphology-regulating lunapark protein induces the formation of stacked bilayer discs. Life Sci Alliance.

[CR32] Wang S, Romano FB, Field CM, Mitchison TJ, Rapoport TA (2013). Multiple mechanisms determine ER network morphology during the cell cycle in Xenopus egg extracts. J Cell Biol.

[CR33] Wang S, Tukachinsky H, Romano FB, Rapoport TA (2016). Cooperation of the ER-shaping proteins atlastin, lunapark, and reticulons to generate a tubular membrane network. Elife.

[CR34] Wang X, Li S, Wang H, Shui W, Hu J (2017). Quantitative proteomics reveal proteins enriched in tubular endoplasmic reticulum of *Saccharomyces cerevisiae*. Elife.

[CR35] Wozniak MJ, Bola B, Brownhill K, Yang YC, Levakova V, Allan VJ (2009). Role of kinesin-1 and cytoplasmic dynein in endoplasmic reticulum movement in VERO cells. J Cell Sci.

[CR36] Wu F, Hu X, Bian X, Liu X, Hu J (2015). Comparison of human and Drosophila atlastin GTPases. Protein Cell.

[CR37] Yan L, Sun S, Wang W, Shi J, Hu X, Wang S, Su D, Rao Z, Hu J, Lou Z (2015). Structures of the yeast dynamin-like GTPase Sey1p provide insight into homotypic ER fusion. J Cell Biol.

[CR38] Zhang H, Hu J (2016). Shaping the endoplasmic reticulum into a social network. Trends Cell Biol.

[CR39] Zhang M, Wu F, Shi J, Zhu Y, Zhu Z, Gong Q, Hu J (2013). ROOT HAIR DEFECTIVE3 family of dynamin-like GTPases mediates homotypic endoplasmic reticulum fusion and is essential for Arabidopsis development. Plant Physiol.

[CR40] Zhao Y, Zhang T, Huo H, Ye Y, Liu Y (2016). Lunapark is a component of a ubiquitin ligase complex localized to the endoplasmic reticulum three-way junctions. j biol chem.

[CR41] Zhu PP, Patterson A, Lavoie B, Stadler J, Shoeb M, Patel R, Blackstone C (2003). Cellular localization, oligomerization, and membrane association of the hereditary spastic paraplegia 3A (SPG3A) protein atlastin. J Biol Chem.

[CR42] Zhu Y, Zhang G, Lin S, Shi J, Zhang H, Hu J (2018). Sec61β facilitates the maintenance of endoplasmic reticulum homeostasis by associating microtubules. Protein Cell.

