# Supplementary material for: Reciprocal regulation between lunapark and atlastin facilitates ER three-way junction formation
Source: Protein Cell. 2018 Nov 29;10(7):510–25. doi: 10.1007/s13238-018-0595-7 (PMC6588657; doi:10.1007/s13238-018-0595-7)
Supplement: Supplementary file 1 — Supplementary material 1 (PDF 3742 kb) [file 13238_2018_595_MOESM1_ESM.pdf]

Figure S1

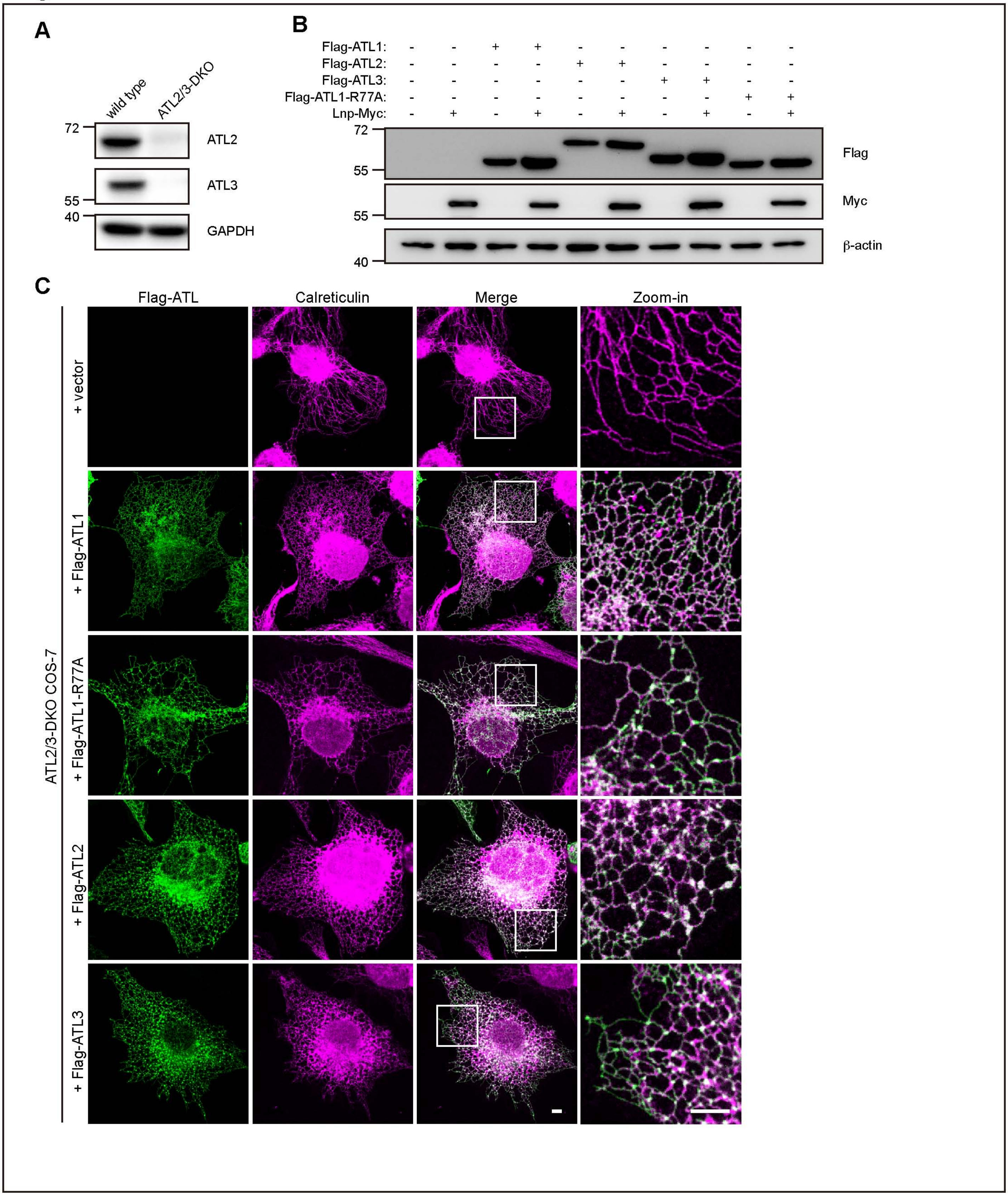
A
wild type
ATL2/3-DKO
72
55
40
ATL2
ATL3
GAPDH
B
Flag-ATL1:
Flag-ATL2:
Flag-ATL3:
Flag-ATL1-R77A:
Lnp-Myc:
72
55
55
40
Flag
Myc
β-actin
C
Flag-ATL
Calreticulin
Merge
Zoom-in
ATL2/3-DKO COS-7
+ vector
+ Flag-ATL1
+ Flag-ATL1-R77A
+ Flag-ATL2
+ Flag-ATL3

Figure S2

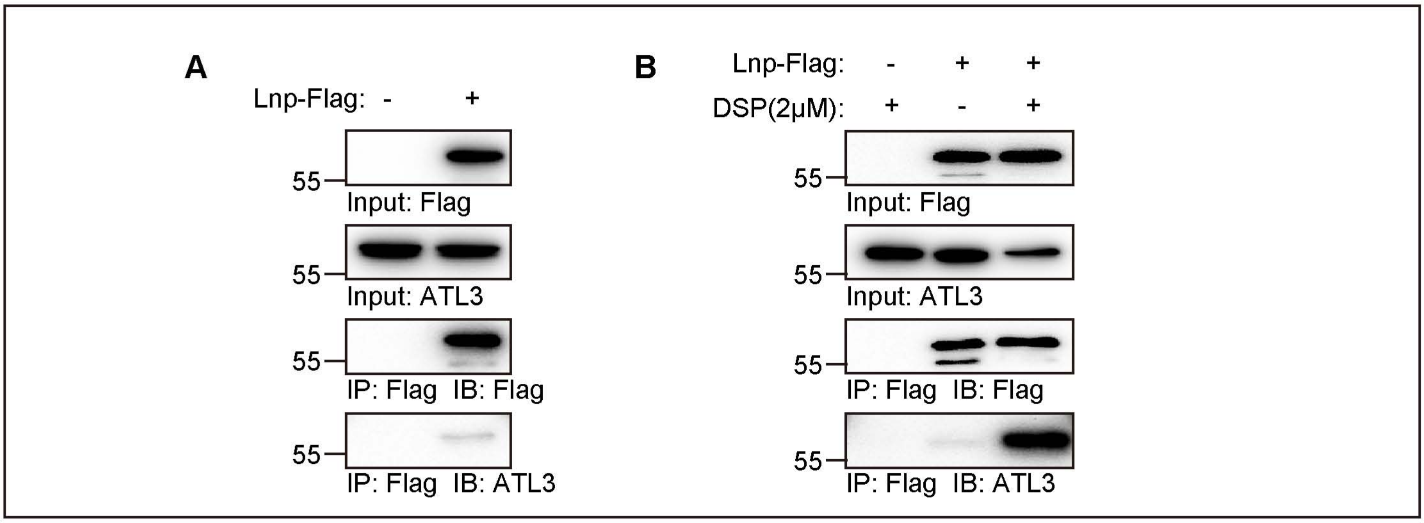
A
Lnp-Flag: - +
55
Input: Flag
55
Input: ATL3
55
IP: Flag IB: Flag
55
IP: Flag IB: ATL3
B
Lnp-Flag: - + +
DSP(2μM): + - +
55
Input: Flag
55
Input: ATL3
55
IP: Flag IB: Flag
55
IP: Flag IB: ATL3

Figure S3

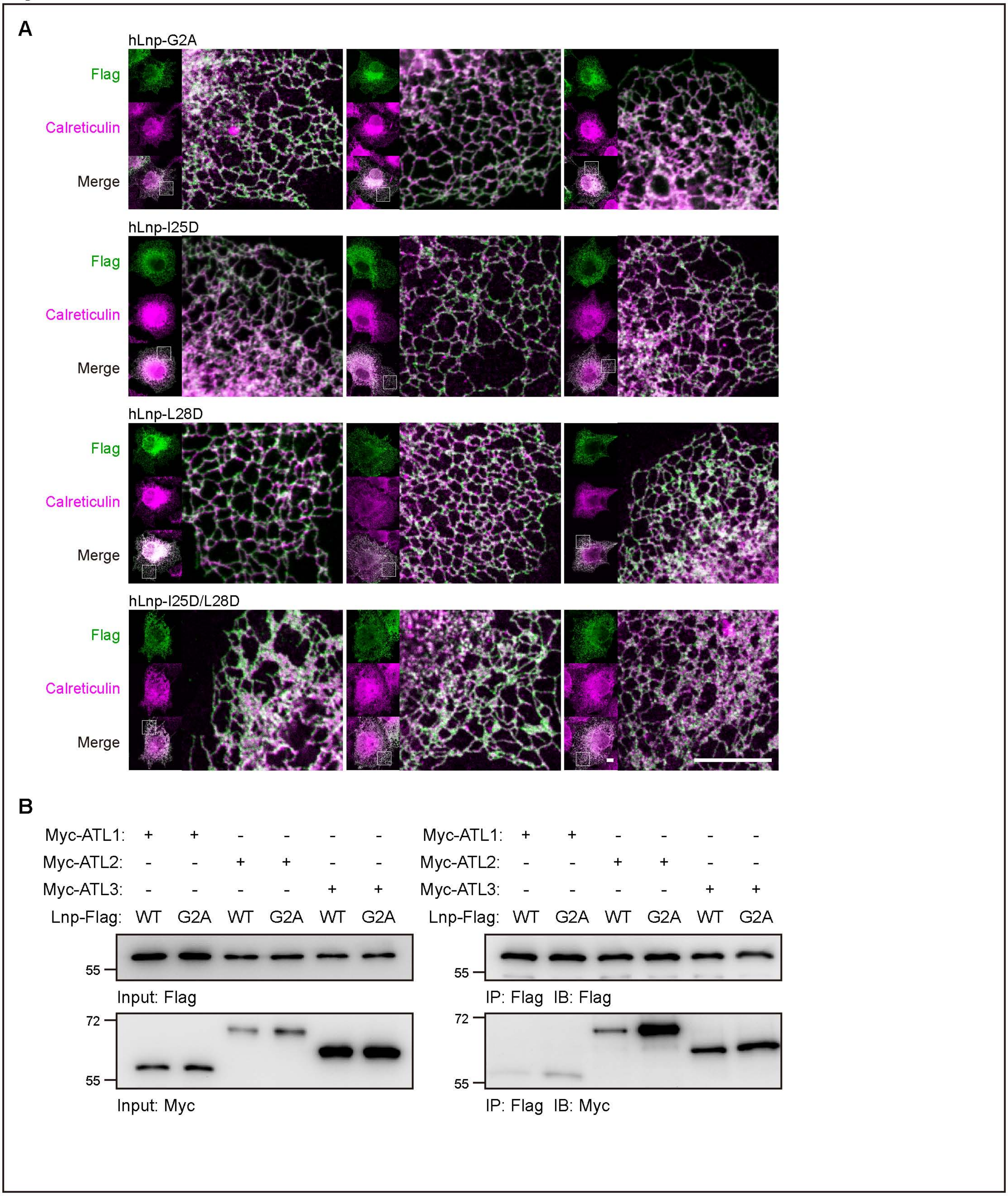
A
hLnp-G2A
Flag
Calreticulin
Merge
hLnp-I25D
Flag
Calreticulin
Merge
hLnp-L28D
Flag
Calreticulin
Merge
hLnp-I25D/L28D
Flag
Calreticulin
Merge
B
Myc-ATL1: + + - - - -
Myc-ATL2: - - + + - -
Myc-ATL3: - - - - + +
Lnp-Flag: WT G2A WT G2A WT G2A
55
Input: Flag
72
55
Input: Myc
Myc-ATL1: + + - - - -
Myc-ATL2: - - + + - -
Myc-ATL3: - - - - + +
Lnp-Flag: WT G2A WT G2A WT G2A
55
IP: Flag IB: Flag
72
55
IP: Flag IB: Myc

Figure S4

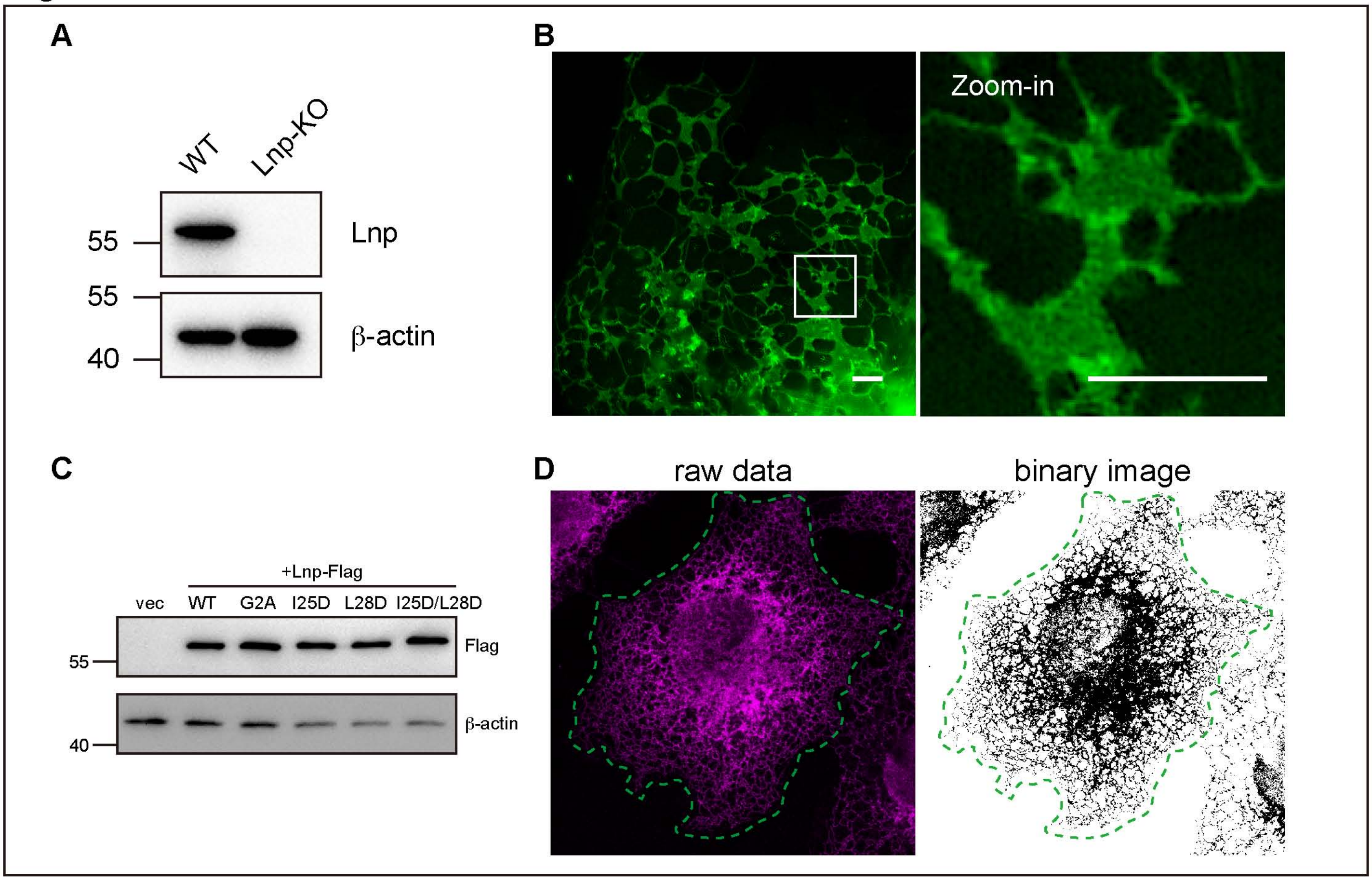
A
WT
Lnp-KO
55
55
40
Lnp
β-actin
B
Zoom-in
C
+Lnp-Flag
vec
WT
G2A
I25D
L28D
I25D/L28D
55
40
Flag
β-actin
D
raw data
binary image

Figure S5

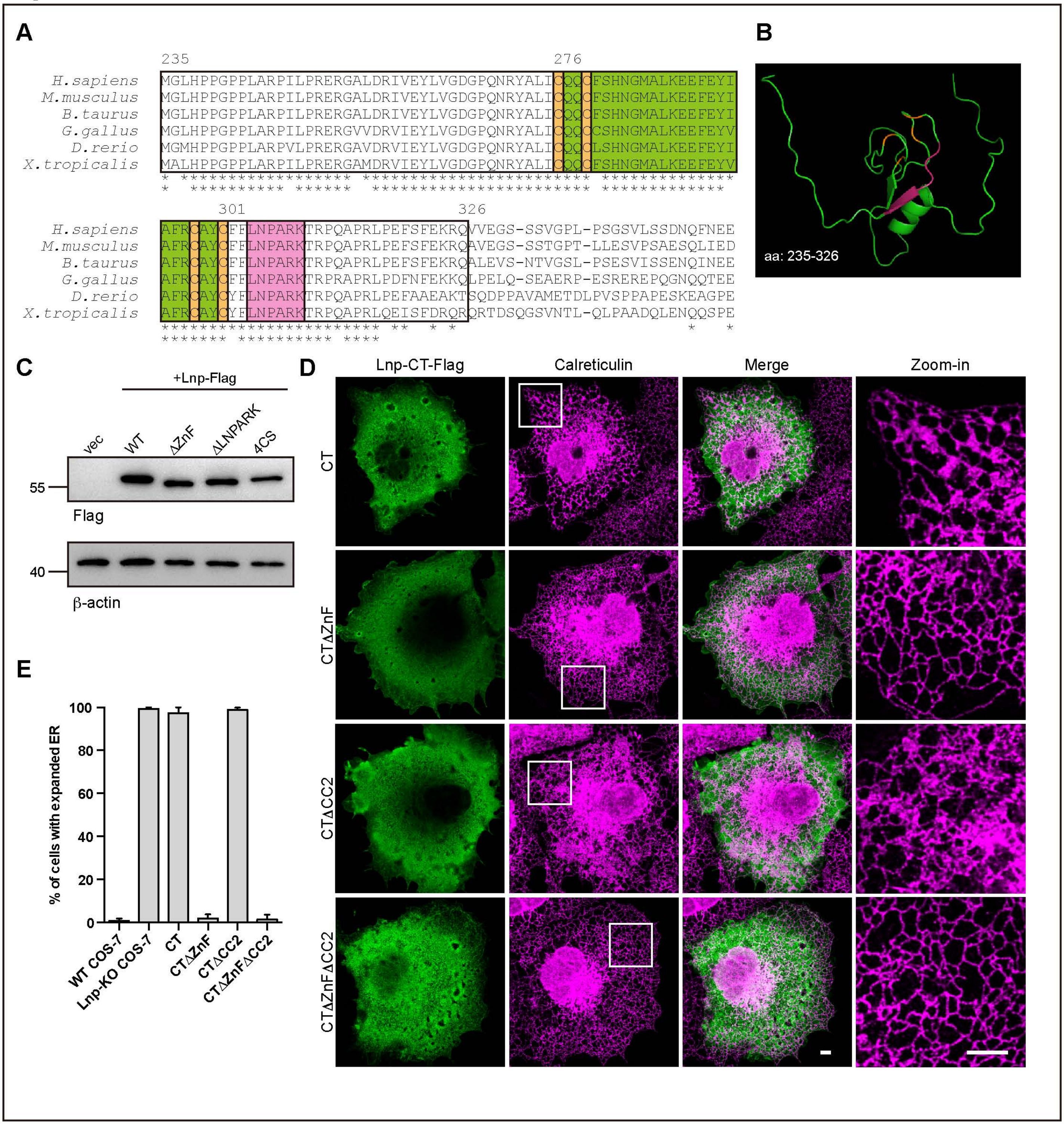
A
235
276
H.sapiens MGLHPPGPPLARPILPRERGALDRIVEYLVGDGPQNRYALICQQCFSHNGMALKEEFEYI
M.musculus MGLHPPGPPLARPILPRERGALDRIVEYLVGDGPQNRYALICQQCFSHNGMALKEEFEYI
B.taurus MGLHPPGPPLARPILPRERGALDRIVEYLVGDGPQNRYALICQQCFSHNGMALKEEFEYI
G.gallus MGLHPPGPPLARPILPRERGVVDRVIEYLVGDGPQNRYALICQQCCSHNGMALKEEFEYV
D.rerio MGMHPPGPPLARPVLPRERGAVDRVIEYLVGDGPQNRYALICQQCLSHNGMALKEEFEYI
X.tropicalis MALHPPGPPLARPILPRERGAMDRVIEYLVGDGPQNRYALICQQCFSHNGMALKEEFEYV
301
326
H.sapiens AFRCAYCFFLNPARKTRPQAPRLPEFSFEKRQVVEGS-SSVGPL-PSGSVLSSDNQFNEE
M.musculus AFRCAYCFFLNPARKTRPQAPRLPEFSFEKRQAVEGS-SSTGPT-LLESVPSAESQLIED
B.taurus AFRCAYCFFLNPARKTRPQAPRLPEFSFEKRQALEVS-NTVGSL-PSESVISSENQINEE
G.gallus AFRCAYCFFLNPARKTRPRAPRLPDFNFEKKQLPELQ-SEAERP-ESREREPQGNQQTEE
D.rerio AFRCAYCYFLNPARKTRPQAPRLPEFAAEAKTSQDPPAVAMETDLPVSPPAPESKEAGPE
X.tropicalis AFRCAYCYFLNPARKTRPQAPRLQEISFDRQRQRTDSQGSVNTL-QLPAADQLENQQSPE
B
aa: 235-326
C
+Lnp-Flag
vec
WT
ΔZnF
ΔLNPARK
4CS
55
Flag
40
β-actin
D
Lnp-CT-Flag
Calreticulin
Merge
Zoom-in
CT
CTΔZnF
CTΔCC2
CTΔZnFΔCC2
E
% of cells with expanded ER
100
80
60
40
20
0
WT COS-7
Lnp-KO COS-7
CT
CTΔZnF
CTΔCC2
CTΔZnFΔCC2

Figure S6

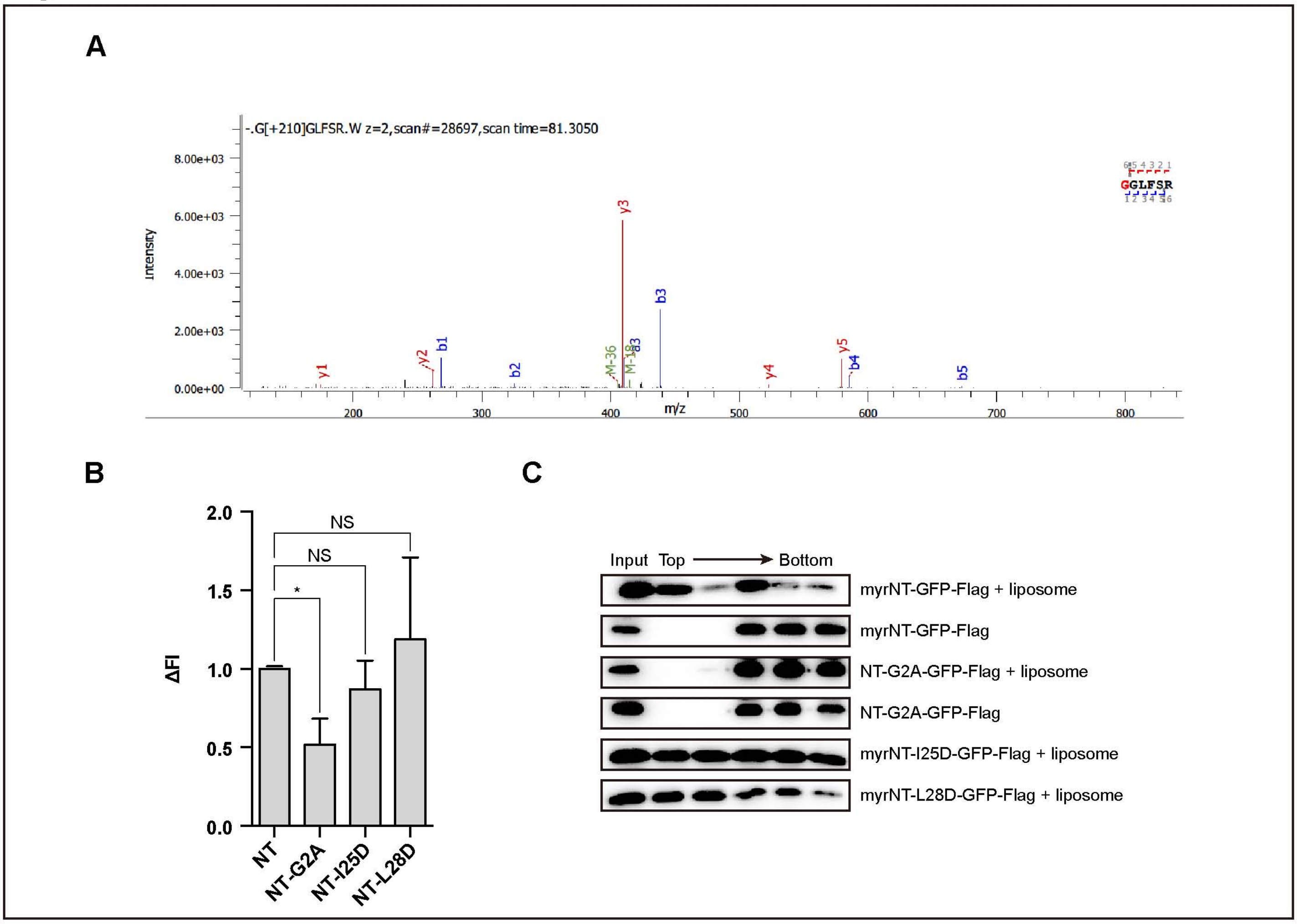
A
-.G[+210]GLFSR.W z=2,scan#=28697,scan time=81.3050
Intensity
m/z
y1
y2
b1
b2
M-36
M-18
a3
y3
b3
y4
y5
b4
b5
GGLFSR
B
ΔFI
NS
NS
*
NT
NT-G2A
NT-I25D
NT-L28D
C
Input
Top
Bottom
myrNT-GFP-Flag + liposome
myrNT-GFP-Flag
NT-G2A-GFP-Flag + liposome
NT-G2A-GFP-Flag
myrNT-I25D-GFP-Flag + liposome
myrNT-L28D-GFP-Flag + liposome

Figure S7

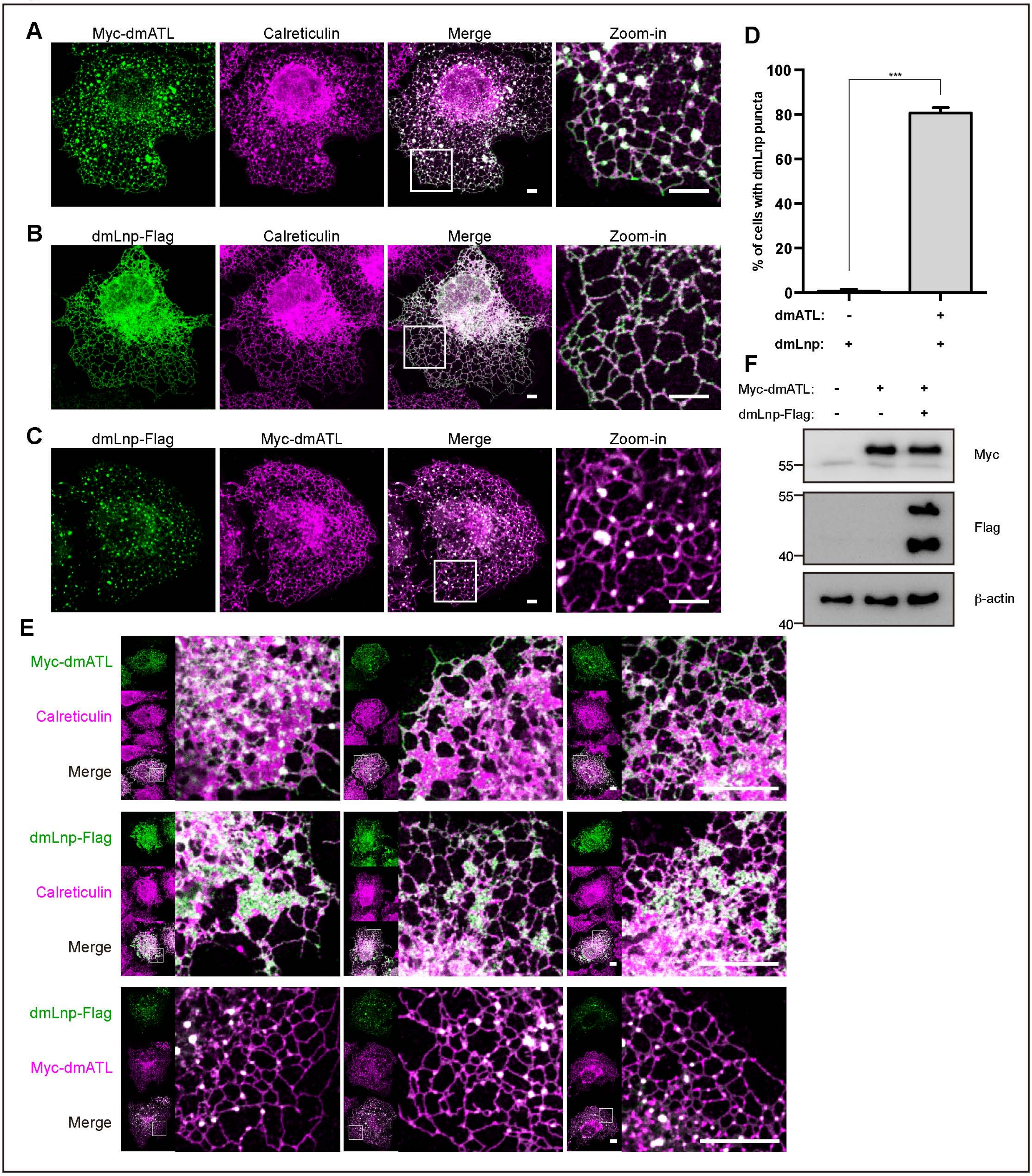
A
Myc-dmATL
Calreticulin
Merge
Zoom-in
B
dmLnp-Flag
Calreticulin
Merge
Zoom-in
C
dmLnp-Flag
Myc-dmATL
Merge
Zoom-in
D
% of cells with dmLnp puncta
100
80
60
40
20
0
***
dmATL: - +
dmLnp: + +
F
Myc-dmATL: - + +
dmLnp-Flag: - - +
55
Myc
55
40
Flag
40
β-actin
E
Myc-dmATL
Calreticulin
Merge
dmLnp-Flag
Calreticulin
Merge
dmLnp-Flag
Myc-dmATL
Merge

Figure S1. Verification of exogenous ATLs and Lnp in ATL-DKO COS-7 cells. (A) Deletion of ATL2 and ATL3 in ATL-deleted (DKO) cells were verified by immunoblotting (IB) using antibodies against ATL2 and ATL3, respectively. GAPDH protein level was measured as a loading control. (B) Expression level of Flag-tagged ATLs and Myc-tagged Lnp in ATL-DKO COS-7 cells were measured by IB using anti-Flag tag and anti-Myc tag antibodies, respectively. β-actin was measured as a loading control. (C) Flag-tagged ATLs (green) were transfected into ATL-DKO COS-7 cells. ER morphology was visualized by indirect immunostaining of endogenous ER luminal marker calreticulin (magenta). Scale bars = 5 μm.

Figure S2. Interactions between endogenous ATL3 and exogenous Lnp. (A) Interaction tests between endogenous ATL3 and Flag-tagged Lnp. Lnp-Flag was transfected in COS-7 cells and precipitated by anti-Flag antibody-conjugated agarose from the cell lysates containing 1% digitonin. Samples were immunoblotted with anti-ATL3 and anti-Flag antibodies. (B) As in (A), but with precipitation in the cell lysates containing 1% Triton X-100. Cells were treated with (lane 3) or without (lane 1 and 2) 2 μM chemical cross-linker DSP (3,3'-dithiodipropionate dithiobis (succinimidyl propionate)) before lysis. Blank vector was used as a negative control in (A) and (B).

Figure S3. **Localization of Lnp with point mutation in the NT.** (A) More examples of indicated mutants of exogenous Flag-tagged Lnp in wild type COS-7 cells. Scale bars = 10 μm. (B) Interaction tests between various Myc-tagged ATLs and Flag-tagged wild-type and G2A Lnp by co-immunoprecipitation. Myc-tagged

ATLs was expressed together with Flag-tagged wild-type LNP (lanes 1, 3, and 5) or the G2A mutant (lanes 2, 4, and 6). Various Lnp-Flag proteins were precipitated from the cell lysates containing 1% Triton X-100 by anti-Flag antibody-conjugated agarose and immunoblotted with anti-Flag and anti-Myc antibodies.

Figure S4. ER expansion in Lnp-KO COS-7 cells. (A) Endogenous Lnp expression levels in wild-type and Lnp-deleted COS-7 cells were investigated by Western blot with anti-Lnp antibodies. β-actin was used as a loading control. (B) High-resolution image of the ER structure in Lnp-deleted COS-7 cells captured using GI-SIM. The ER was visualized by mEmerald-labeled calreticulin. The right panel shows the 8× enlargement of the area of interest. Scale bars = 2.5 μm. (C) Expression level of exogenous Flag-tagged Lnp in Lnp-KO COS-7 cells were measured by western blot using anti-Flag antibody. β-actin protein level was used as a loading control. (D) Measurement of the ER area. Before calculating the ER area, image of calreticulin (left) was transformed into RenyiEntropy binary image (right) in Image J. Area of interested cell (surrounded by green dash line) and its ER area (in black) were measured and calculated by Image J.

Figure S5. Dominant-negative effect of Lnp-CT through its conserved ZnF. (A) Sequence alignment of the zinc finger and its flanking regions in Lnp from the indicated species. The C4-type zinc finger (residues 276-301) is highlighted in green. Four cysteine residues are highlighted in yellow. The LNPARK motif is highlighted in pink. *, similar residues; **, identical residues. (B) Molecular model of the fragment (residues 236-326) emphasized in the black box in (A)

generated using the online program RaptorX (http://raptorx.uchicago.edu/StructurePrediction/predict/). The positions of the cysteine residues and LNPARK motif are shown in orange and pink, respectively. (C) Expression level of wild-type and various Flag-tagged Lnp mutants in Lnp-KO COS-7 cells were measured by western blot using anti-Flag tag antibody. β-actin protein level was used as a loading control. (D) The indicated Flag-tagged Lnp-CT constructs were expressed in wild-type COS-7 cells. Their localization was investigated using anti-Flag antibodies (green). ER morphology was visualized by indirect immunostaining of endogenous ER luminal marker calreticulin (magenta). Scale bars = 5 μm. (E) Percentage of cells with expanded ER in (D). Columns are shown in Mean ± SD. A total of 50 to 60 cells were categorized for each sample. Cell numbers were counted in three independent experiments.

Figure S6. *N*-myristoylation conveys the majority of lipid affinity. (A) *N*-myristoylation of purified Lnp-NT was verified by mass spectrometry. The myristoyl group is shown as its molecular weight (+210) at G2. (B) Measurement of the FRET signal between the indicated GFP-tagged proteins and rhodamine-labeled liposomes. Data are normalized to wild-type NT and presented as mean ± SEM. *P < 0.05; NS, no significance by one-tailed non-parametric Mann-Whitney test. Experiments were performed three times. (C) Proteoliposomes containing the indicated wild-type Lnp-NT or mutants were floated in a sucrose gradient and the fractions analyzed by immunoblotting with anti-Flag antibody.

Figure S7. **Localization of ectopically expressed dmLnp depends on dmATL.** (A) Myc-tagged drosophila dmATL was expressed in COS-7 cells. Localization of dmATL was determined by anti-Myc antibody (green). The ER was visualized by indirect immunostaining of endogenous ER luminal marker calreticulin (magenta). (B) As in (A), but with Flag-tagged drosophila dmLnp expressed in COS-7 cells. (C) Flag-tagged dmLnp was co-expressed with Myc-tagged dmATL in COS-7 cells. Their localization was determined by anti-Flag (green) and anti-Myc antibodies (magenta). Scale bars = 5 μm. (D) Percentage of cells with dmLnp-enriched puncta in (B) and (C). Columns are shown in Mean ± SD. A total of 50 to 60 cells were categorized for each sample. Cell numbers were counted in three independent experiments. ***, $P < 0.001$ by Fisher's exact test. (E) Myc-dmATL, or dmLnp-Flag, or both were transfected into Lnp-deleted COS-7 cells. Protein localization and ER morphology were visualized by indirect immunostaining using indicated antibodies. (F) Expression Flag-tagged dmLnp and Myc-tagged dmATL in ATL-DKO COS-7 cells were verified by western blot using anti-Flag tag and anti-Myc tag antibodies, respectively. β-actin protein level was used as a loading control.
